# Nootkatone Derivative Nootkatone-(E)-2-iodobenzoyl hydrazone Promotes Megakaryocytic Differentiation in Erythroleukemia by Targeting JAK2 and Enhancing JAK2/STAT3 and PKCδ/MAPK Crosstalk

**DOI:** 10.3390/cells14010010

**Published:** 2024-12-26

**Authors:** Yang Pan, Feng Xiao, Chaolan Pan, Hui Song, Peng Zhao, Meijun Chen, Liejun Huang, Jue Yang, Xiaojiang Hao

**Affiliations:** 1 State Key Laboratory of Functions and Applications of Medicinal Plants, Guizhou Medical University, Guiyang 550014, China; deer7813py@163.com (Y.P.); xxiaofeng1026@163.com (F.X.); panchaolan202312@163.com (C.P.); zhaopeng960528@163.com (P.Z.); chenmeijunnn@163.com (M.C.); huangliejun@126.com (L.H.); 2 Natural Products Research Center of Guizhou Province, Guiyang 550014, China; 3 Key Laboratory of Endemic and Ethnic Diseases, Ministry of Education, Key Laboratory of Medical Molecular Biology of Guizhou Province, Guizhou Medical University, Guiyang 550044, China; songhui620@gmc.edu.cn; 4 School of Pharmaceutical Sciences, Guizhou Medical University, Guiyang 561113, China; 5 State Key Laboratory of Phytochemistry and Plant Resources in West China, Kunming Institute of Botany, Chinese Academy of Sciences, Kunming 650204, China

**Keywords:** erythroleukemia, nootkatone derivatives, megakaryocytic differentiation, JAK2, JAK2/STAT3, PKCδ/MAPK

## Abstract

Erythroleukemia, a complex myeloproliferative disorder presenting as acute or chronic, is characterized by aberrant proliferation and differentiation of erythroid cells. Although nootkatone, a sesquiterpene derived from grapefruit peel and Alaska yellow cedar, has shown anticancer activity predominantly in solid tumors, its effects in erythroleukemia remain unexplored. This study aimed to investigate the impact of nootkatone and its derivatives on erythroleukemia. Our results demonstrate that the nootkatone derivative nootkatone-(E)-2-iodobenzoyl hydrazone (N2) significantly inhibited erythroleukemia cell proliferation in a concentration- and time-dependent manner. More importantly, N2 induced megakaryocytic differentiation, as evidenced by significant morphological changes, and upregulation of megakaryocytic markers CD41 and CD61. In vivo, N2 treatment led to a marked increase in platelet counts and megakaryocytic cell counts. Mechanistically, N2 activated a crosstalk between the JAK2/STAT3 and PKCδ/MAPK signaling pathways, enhancing transcriptional regulation of key factors like *GATA1* and *FOS*. Network pharmacology and experimental validation confirmed that N2 targeted JAK2, and knockdown of JAK2 abolished N2-induced megakaryocytic differentiation, underscoring JAK2’s critical role in erythroleukemia differentiation. In conclusion, N2 shows great promise as a differentiation therapy for erythroleukemia, offering a novel approach by targeting JAK2-mediated signaling pathways to induce megakaryocytic differentiation.

## 1. Introduction

Acute myeloid leukemia (AML) is predominantly found in adults, though it also affects children, posing a significant clinical burden with high incidence rates [1]. A specific form of AML, acute erythroleukemia (AEL), also known as AML-M6, originates from the erythroid lineage and accounts for approximately 3–5% of all AML cases. AEL is inherently associated with poor prognostic outcomes and typically shows limited responsiveness to standard therapies like hypomethylating agents and intensified chemotherapy, leaving bone marrow transplantation as the only potentially curative treatment option [2,3]. However, financial and logistical barriers, including high costs and the challenge of finding compatible donors, limit the accessibility of this treatment. Given that AEL arises from megakaryocyte-erythrocyte progenitors (MEPs), which have the potential to differentiate into either erythroid or megakaryocytic cells, research has focused on therapeutic agents that could induce differentiation along these pathways [4].

Recent advances highlight the importance of natural compounds and their derivatives as sources for anticancer drugs, with approximately 50% of small-molecule drugs between 1981 and 2019 being derived from natural products or their synthetic variants [5]. In AEL, several natural compounds have shown promise as differentiation-inducing agents. For instance, 6,7-dimethoxycoumarin promotes the erythroid differentiation of K562 cells via activating FOXO3/p27 signaling pathway [6]. Another compound, nobiletin, a polymethoxyflavone, has demonstrated effectiveness in inducing megakaryocytic differentiation in erythroleukemia cells via activation of the MAPK/ERK pathway to upregulate EGR1 expression [7]. Our research group has previously shown that kaempferol-3-O-α-L-(4″-E-p-coumaroyl) rhamnoside, isolated from Cyclocarya paliurus leaves, can promote megakaryocytic differentiation and inhibit leukemic proliferation in erythroleukemia models through the PKCδ/ERK1/2 pathway [8]. Despite these developments, no differentiation-inducing drugs for AEL are currently available in clinical practice, highlighting the urgent need for novel therapeutic agents.

Nootkatone, a sesquiterpene commonly isolated from grapefruit peel and Alaska yellow cedar trees, has a unique flavor and safety profile, which has led to its broad application in the food industry [9]. Beyond its aromatic qualities, nootkatone exhibits a range of biological activities, including anti-inflammatory [10,11], antibacterial [12], insect-repellent [13,14], neuroprotective [15,16], and anti-tumor properties [17,18,19,20]. Prior studies have demonstrated nootkatone’s efficacy against non-small-cell lung cancer, colorectal cancer, and retinoblastoma, indicating potential anticancer properties across diverse cancer types [17,18,19]. Cho et al. showed that nootkatone impairs glucose metabolism by targeting AMPK, reducing stemness of breast cancer stem cells, suggesting a possible role in influencing cancer cell differentiation [20]. However, no studies to date have explored the potential of nootkatone in leukemia, particularly in erythroleukemia.

In response to this knowledge gap, our research team synthesized a series of nootkatone derivatives, including a promising derivative, nootkatone-(E)-2-iodobenzoyl hydrazone (N2), through structural modifications [21]. The present study examines the anti-erythroleukemia activity of N2, specifically its ability to induce megakaryocytic differentiation through in vitro and in vivo models. Mechanistic studies suggest that N2′s activity may be mediated by dual activation of JAK2/STAT3 and PKCδ/MAPK signaling via targeting JAK2, representing a novel mechanism of action in differentiation therapy for AEL.

## 2. Materials and Methods

### 2.1. Cell Culture

Human erythroleukemia cell lines HEL and K562 were obtained from the American Type Culture Collection (ATCC, Manassas, VA, USA). HEL cells were cultured in RPMI 1640 medium (Gibco Laboratories, Grand Island, NY, USA), while K562 cells were maintained in Dulbecco’s Modified Eagle’s Medium (DMEM, Gibco Laboratories), both supplemented with 10% fetal bovine serum (FBS, Gibco Laboratories) and 1% penicillin-streptomycin (New Cell and Molecular Biotech, Suzhou, China). All cell cultures were incubated in a humidified atmosphere with 5% CO_2_ at 37 °C.

### 2.2. MTT Assay

To evaluate N2′s effects on cell viability, HEL (1 × 10^4^ cells per well) and K562 (8 × 10^3^ cells per well) were seeded into 96-well plates and treated with various concentrations of N2 (0.625 μM to 20 μM) over designated time intervals. A 0.1% DMSO group was a negative control group. Following treatment, 10 μL of MTT solution (Solarbio, Beijing, China) was added to each well, and the optical density (OD) was measured at 570 nm using a multiwell spectrophotometer (BioTek, Winooski, VT, USA).

### 2.3. Wright–Giemsa Staining

HEL and K562 cells were treated with N2, JAK2 inhibitor WP1066 (1 μM, MCE, Monmouth Junction, NJ, USA) and PKCδ inhibitor Rottlerin (1 μM, GlpBio, San Diego, CA, USA) for the specified durations; 0.1% DMSO group was a negative control group. The treated cells were cytocentrifuged (Shandon Cytospin4, Thermo Fisher Scientific, Waltham, MA, USA) onto microscope slides and then subjected to Wright–Giemsa solution (Solarbio, Beijing, China) for 1–2 min. An equal volume of 0.01 M phosphate buffer (pH 6.4–6.8) was added for 3–5 min, followed by rinsing with distilled water. The stained cells were then examined under a light microscope (Olympus CKX53, Tokyo, Japan), and cell images were documented at 400× magnification to capture differentiation-related morphological features.

### 2.4. Cell Cycle and Ploidy Analysis

Cell cycle and ploidy analysis were performed using a FACSCalibur flow cytometer (BD Biosciences, Franklin Lakes, NJ, USA). Briefly, cells were fixed in 70% ethanol at −20 °C overnight and stained with propidium iodide (PI, BD Biosciences) for 30 min at 37 °C in the dark. For ploidy analysis, cellular debris was excluded by gating based on forward scatter (FSC) and side scatter (SSC) parameters, and a total of 10,000 live-cell events were collected within this gate for each sample and analyzed for DNA content using the PI histogram. For cell cycle analysis, additional gating strategies were employed: (A) FSC vs. SSC to exclude debris, (B) PE height vs. PE area to eliminate doublets, and (C) the gated live-cell population was applied to the PI histogram to determine the distribution of cell cycle phases. A 0.1% DMSO group was a negative control group.

### 2.5. Cell Differentiation Analysis

For cell differentiation analysis, the expression of megakaryocyte-specific markers CD41a and CD61 was evaluated by flow cytometry. After treatment with N2, WP1066 (1 μM) and Rottlerin (1 μM) for 72 h, HEL and K562 cells were incubated with FITC- or APC-conjugated anti-CD41a (1:200) or APC-conjugated anti-CD61 (1:200) (BD Biosciences, Franklin Lakes, NJ, USA) for 30 min on ice in the dark, followed by immediate analysis using a FACSCalibur flow cytometer. A 0.1% DMSO group was a negative control group.

### 2.6. RNA Isolation and qRT-PCR Analysis

RNA extraction was performed on HEL and K562 cells treated with different concentrations of N2 for 48 h using Trizol reagent (Invitrogen, Carlsbad, CA, USA). cDNA synthesis was carried out using the PrimeScript RT reagent kit with gDNA Eraser (TaKaRa, Dalian, China). Quantitative real-time PCR (qRT-PCR) was performed using specific primers and SYBR Green Master Mix on the LightCycler 480 System (Roche, Mannheim, Germany), with GAPDH as a housekeeping control. A 0.1% DMSO group was a negative control group. Primer sequences are provided in Table S1.

### 2.7. Western Blotting

HEL and K562 cells were treated with N2, WP1066 (1 μM), and Rottlerin (1 μM) for 72 h. A 0.1% DMSO group was a negative control group. Cells were harvested, lysed, and analyzed by Western blotting, as described previously [22]. Membranes were probed with primary antibodies specific to GAPDH, GATA1, JAK2, p-JAK2, STAT3, p-STAT3, PKCδ, p-PKCδ, MEK, p-MEK, ERK, and p-ERK overnight at 4 °C. Following incubation with secondary antibodies for 2 h at room temperature, blots were visualized using an Odyssey imaging system (LI-COR Biosciences, Lincoln, NE, USA). GAPDH was used as the loading control. The details of antibodies used in the Western blotting experiments are provided in Table S2.

### 2.8. Immunofluorescence Analysis

HEL and K562 cells were cultured in 12-well plates and treated with N2 at concentrations of 2 μM, 4 μM, and 8 μM for 72 h. A 0.1% DMSO group was a negative control group. After treatment, cells were fixed with paraformaldehyde (Solarbio, Beijing, China) for 15 min at room temperature, washed with PBS, and incubated with anti-CD41a-FITC (1:200) (BD Biosciences) for 2 h at 4 °C. Nuclei were counterstained with DAPI (Solarbio). After washing, images were acquired using a fluorescence microscope (Leica DMi8, Wetzlar, Germany).

### 2.9. Network Pharmacology Analysis

The potential targets of N2 were predicted following previous methodology [23]. In brief, N2 targets were identified using SwissTargetPrediction (http://www.swisstargetprediction.ch/, accessed on 25 October 2023) and intersected with AML-related disease targets from databases (CTD, DISEASES, MalaCards, and DisGeNET) using a Venn diagram.

### 2.10. Molecular Docking Analysis

The 3D crystal structure of JAK2 was obtained from the Protein Data Bank (PDB) (https://www.rcsb.org/, accessed on 28 August 2024). Molecular docking was performed using AutoDock Vina 1.1.2 (The Scripps Research Institute, La Jolla, CA, USA), and the interaction between N2 and JAK2 was analyzed using PLIP v2.3.0 (https://plip-tool.biotec.tu-dresden.de/plip-web/plip/index, accessed on 9 September 2024) [24]. The docking results were visualized using PyMOL (Version 3.1.0a0).

### 2.11. Cellular Thermal Shift Assay (CETSA)

HEL cells were lysed in PBS buffer containing 1 mM protease inhibitor cocktail (Solarbio, Beijing, China) via three freeze–thaw cycles in liquid nitrogen. The supernatant was divided into two portions and treated with N2 (20 μM) or 0.1% DMSO for 1 h at room temperature. Equal protein amounts were subjected to a temperature gradient (40 °C to 52 °C) for 3 min. After centrifugation, samples were analyzed by Western blotting.

### 2.12. Drug Affinity Responsive Target Stability (DARTS)

HEL cells were lysed in RIPA buffer (Solarbio, Beijing, China). The supernatant was divided into three portions and incubated with different concentrations of N2 (10 μM, 20 μM) or 0.1% DMSO for 1 h at room temperature. Samples were then treated with varying concentrations of pronase (Roche, Mannheim, Germany) for 30 min at room temperature, followed by Western blot analysis.

### 2.13. Lentiviral Knockdown and Stable Cell Line Generation

JAK2 knockdown lentiviruses (LV-sh-JAK2) were purchased from Genechem (Shanghai, China). HEL cells were infected with JAK2 knockdown or control lentiviruses for 72 h. Stable cell lines were selected using puromycin (Solarbio, Beijing, China), and JAK2 knockdown efficiency was confirmed by Western blotting. The target sequence for JAK2: NO.1: TAGCTCATTAAGGGAAGCTTT, NO.2: AAGCAACTGTCATGGCCCAAT, NO.3: CTGCAGTACACATCTCAGATA.

### 2.14. In Vivo Experiments

An allograft erythroleukemia mouse model was developed using murine erythroleukemia cells (CB3, 8 × 10⁵) injected via the tail vein into BALB/c mice. Starting from the second day of CB3 injection, N2 was administered intraperitoneally (10 mg/kg or 20 mg/kg) every other day for 21 days. Hematocrit levels, platelet counts, and spleen weights were recorded. Hematoxylin and eosin (H&E, Beyotime, Shanghai, China) staining was performed to evaluate tissue morphology and toxicity on spleen, heart, liver, lung, and kidney, and examined under a light microscope (Olympus CKX53, Tokyo, Japan). Megakaryocytic markers CD41 and CD61 of spleen were analyzed by flow cytometry. Blood chemistry, including total bilirubin (TBIL), creatinine (CRE), blood urea nitrogen (BUN), glutamic oxaloacetic transaminase (GOT) and glutamic pyruvic transaminase (GPT) were assessed using specific detection kits (Jiancheng, Nanjing, China). All experimental procedures were approved by the Animal Care and Use Committee of Guizhou Medical University (Approval No. 2304545).

### 2.15. Flow Cytometry Analysis of Spleen Cells

Single-cell suspensions were prepared by grinding spleens and filtering the homogenate, followed by red blood cell removal using Red Blood Cell Lysis Solution (Solarbio, Beijing, China). The resulting splenocytes were stained with FITC-conjugated anti-CD41 (1:100) or FITC-conjugated anti-CD61 antibodies (1:100) (BD Biosciences, Franklin Lakes, NJ, USA) for 30 min on ice in the dark. After staining, the cells were immediately analyzed using a FACSCalibur flow cytometer (BD Biosciences). Flow cytometry gating was performed to ensure accurate analysis: viable singlet populations were selected using FSC and SSC parameters to exclude debris and doublets. From this viable cell gate, a total of 10,000 events were collected for each sample. Positive intervals were determined using isotype controls, and quadrant gates were established based on the boundaries of the positive interval. The proportions of CD41^+^ and CD61^+^ cells were subsequently quantified within the pre-gated population.

### 2.16. Statistical Analysis

All data are presented as mean ± standard deviation (SD) from a minimum of three independent experiments. Statistical significance between two groups was assessed using a two-tailed unpaired Student’s *t*-test. For multiple group comparisons, one-way analysis of variance (ANOVA) was employed. A *p*-value of less than 0.05 was considered statistically significant.

## 3. Results

### 3.1. Nootkatone Derivative N2 Inhibits Cell Proliferation in Erythroleukemia HEL and K562 Cells

**Figure 1:**
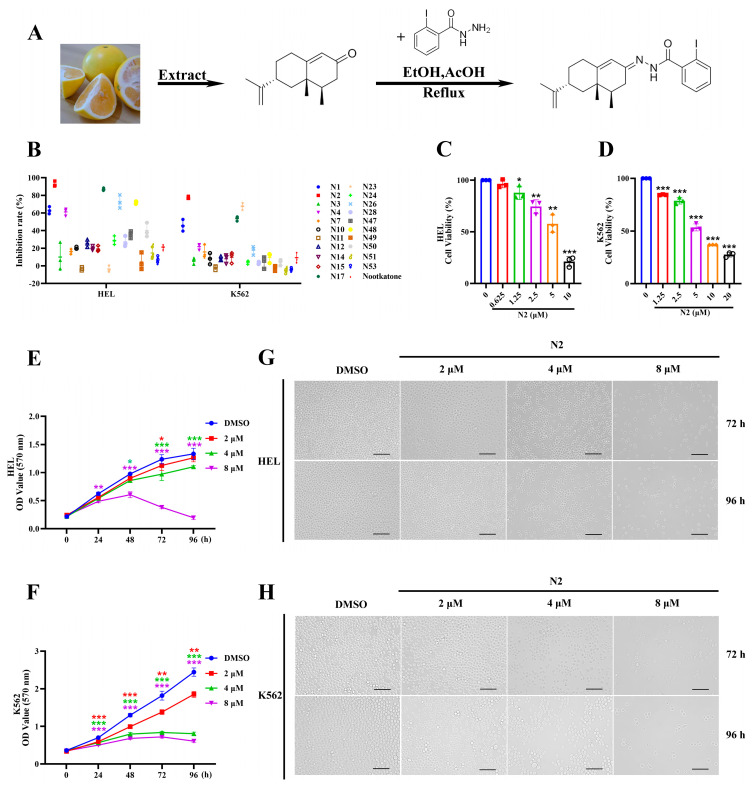
Nootkatone derivative N2 inhibits cell proliferation in erythroleukemia HEL and K562 cells. (**A**) Chemical structure of nootkatone derivative N2. (**B**) Scatter diagram presentation of the influence of nootkatone and its derivatives (20 μM) on cell viability of HEL and K562 cells for 72 h. The inhibition rate was measured by MTT assays, with the 0.1% DMSO group serving as the negative control. Inhibition rate = (negative control group—treatment group)/negative control group × 100%. (**C**,**D**) HEL and K562 cells were treated with varying concentrations of the nootkatone derivative N2 for 72 h, and cell viability was evaluated using MTT assays. (**E**,**F**) The influence of N2 on the proliferation of HEL and K562 cells was quantified through MTT assays. (**G**,**H**) Effects of N2 on morphological changes in HEL and K562 cells. Magnification: ×200. Scale bar: 100 µm. Data represented the mean ± SD of three independent experiments. * *p* < 0.05, ** *p* < 0.01, *** *p* < 0.001 vs. the DMSO group.

To assess the anti-proliferative activity of nootkatone and its derivatives, HEL and K562 cells were treated with varying concentrations of these compounds for 72 h. MTT assay results demonstrated that the nootkatone derivative N2 (Figure 1A) significantly reduced cell viability in a dose-dependent manner in both HEL (Figure 1B,C) and K562 (Figure 1B,D) cells. The IC_50_ values were calculated as 4.58 ± 0.15 µM for HEL cells and 6.54 ± 0.27 µM for K562 cells (Table S3). The growth curve analysis further confirmed that N2 exerted a marked inhibitory effect on HEL and K562 cells’ proliferation in a time- and dose-dependent manner (Figure 1E,F). Microscopic examination of cells treated with N2 (2, 4, and 8 µM) for 72–96 h revealed dose-dependent morphological changes, including increased cell size and attachment in both HEL (Figure 1G) and K562 (Figure 1H) cells compared to controls.

### 3.2. Nootkatone Derivative N2 Promotes Megakaryocytic Differentiation of HEL and K562 Cells

**Figure 2:**
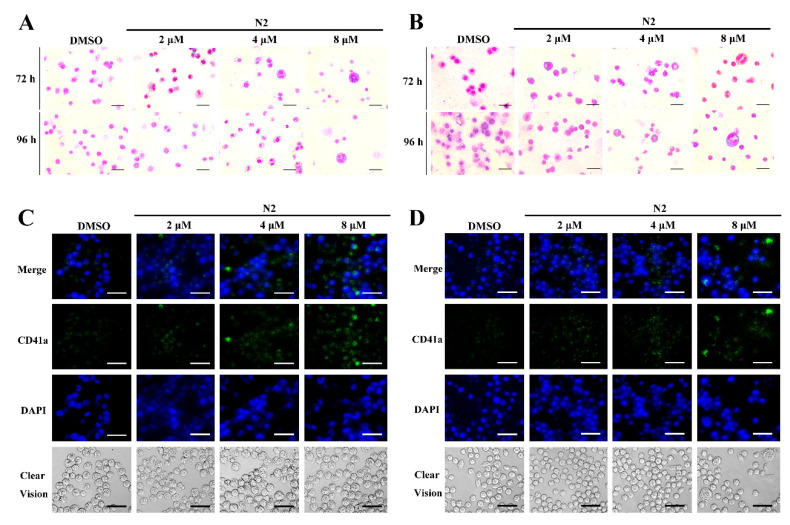
Nootkatone derivative N2 enhances multinucleation and CD41a expression in HEL and K562 cells. (**A**,**B**) Morphological analysis of HEL (**A**) and K562 (**B**) cells following treatment with varying concentrations of N2 for 72–96 h. Cells were subjected to Wright–Giemsa staining for morphological assessment. Magnification: ×400. Scale bar: 50 µm. (**C**,**D**) Immunofluorescence analysis was conducted on HEL (**C**) and K562 (**D**) cells treated with either DMSO or N2 for 72 h, revealing CD41a expression (green). Nuclear staining was performed using DAPI (blue). Magnification: ×400. Scale bar: 50 µm.

**Figure 3:**
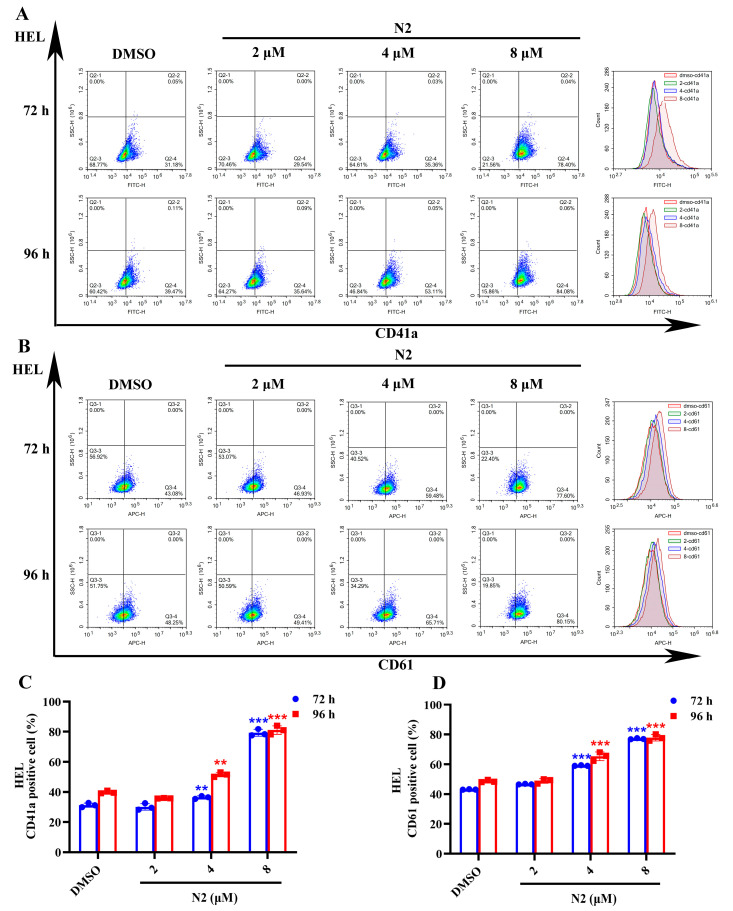
Nootkatone derivative N2 increases the expression of megakaryocyte-specific markers in HEL cells. (**A,B**) Expression of CD41a and CD61 megakaryocyte-specific markers analyzed using flow cytometry in HEL cells. (**C**,**D**) Quantification of the percentage of CD41a^+^ cells and CD61^+^ cells in HEL cells. Data represent the mean ± SD of three independent experiments. ** *p* < 0.01, *** *p* < 0.001 vs. the DMSO group.

**Figure 4:**
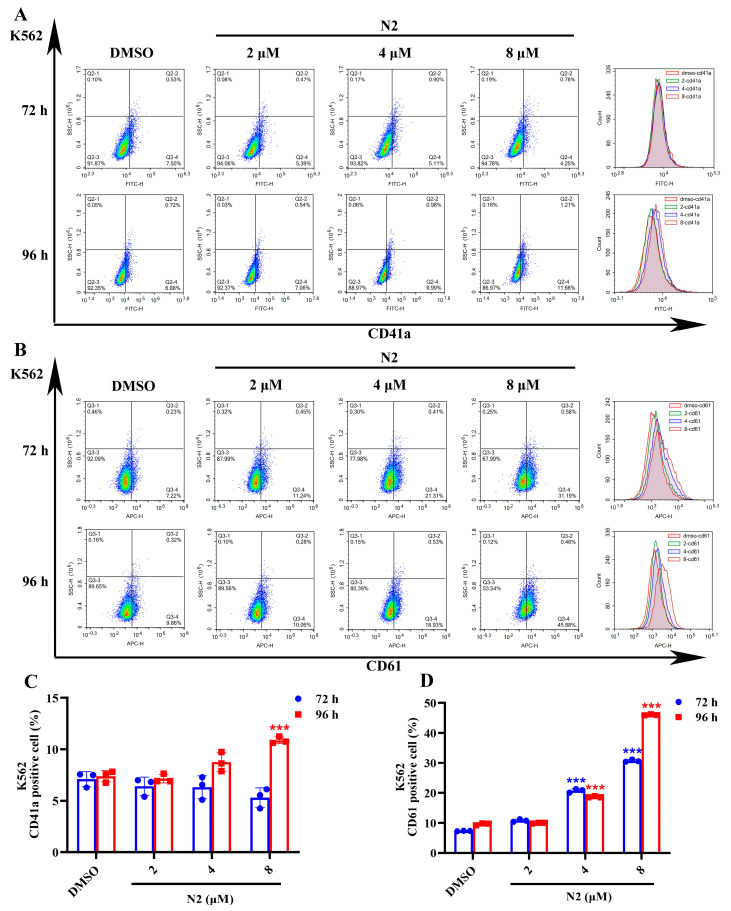
Nootkatone derivative N2 increases the expression of megakaryocyte-specific markers in K562 cells. (**A**,**B**) Expression of CD41a and CD61 megakaryocyte-specific markers analyzed using flow cytometry in K562 cells. (**C**,**D**) Quantification of the percentage of CD41a^+^ cells and CD61^+^ cells in K562 cells. Data represent the mean ± SD of three independent experiments. *** *p* < 0.001 vs. the DMSO group.

Microscopic analysis showed that N2 treatment induced the appearance of larger, megakaryocyte-like cells (Figure 1G,H). To confirm the differentiation-inducing effect of N2, Wright–Giemsa staining, immunofluorescence, and flow cytometry were performed. Wright–Giemsa staining indicated that the N2-treated cells exhibited multinucleation, a hallmark of megakaryocytic differentiation, in both HEL (Figure 2A) and K562 (Figure 2B) cells, whereas the control group displayed lower levels of multinucleation. Immunofluorescent staining revealed increased expression of the megakaryocytic marker CD41a in a dose-dependent manner in HEL (Figure 2C) and K562 (Figure 2D) cells. Flow cytometry further confirmed the elevated expression of CD41a and CD61, with a significant increase in the percentage of positive cells in the N2-treated groups compared to controls (Figure 3 and Figure 4). These results suggest that N2 promotes megakaryocytic differentiation in HEL and K562 cells.

### 3.3. Effect of Nootkatone Derivative N2 on Cell Cycle Distribution in HEL and K562 Cells

**Figure 5:**
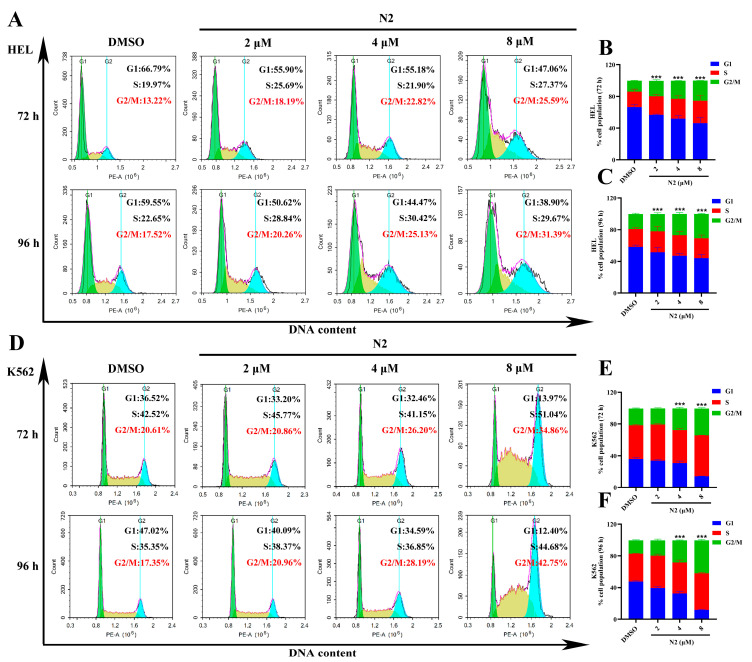
Effect of nootkatone derivative N2 on cell cycle distribution in HEL and K562 cells. (**A**–**F**) HEL (**A**) and K562 (**D**) cells were exposed to indicated concentrations of N2 for either 72 h or 96 h. The cells were stained with PI and the percentage of cell cycle distribution was analyzed by flow cytometry. The proportions of cell cycle distribution at G1, S, and G2 phases in HEL (**B**,**C**) and K562 (**E**,**F**) cells at various time points. Data are expressed as the mean ± SD, with each experiment conducted in triplicate. *** *p* < 0.001 vs. the DMSO group.

**Figure 6:**
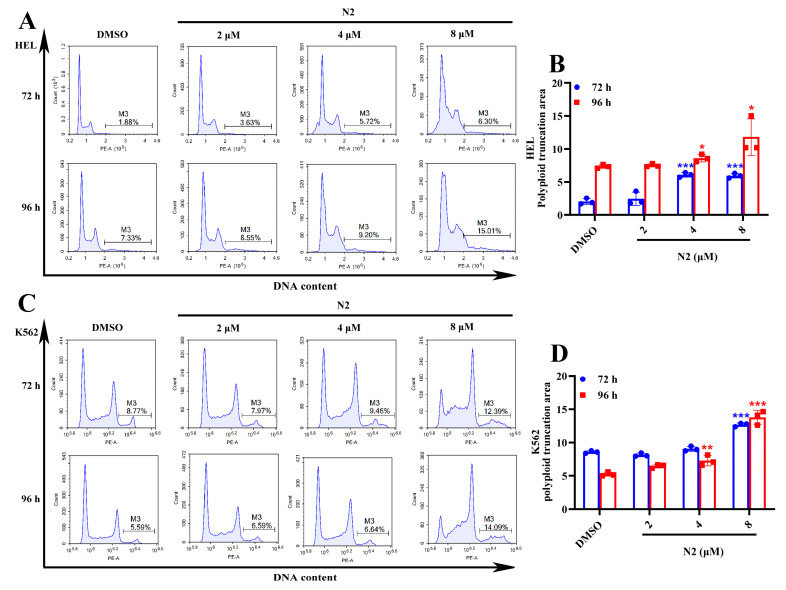
N2 treatment was associated with an increase in polyploidy in HEL and K562 cells. (**A**–**D**) Polyploid cells in N2-treated HEL (**A**) and K562 (**C**) cells were analyzed by flow cytometry. The proportion of polyploid cells in HEL (**B**) and K562 (**D**) cells. Data are expressed as the mean ± SD. Each experiment was repeated in triplicate. * *p* < 0.05, ** *p* < 0.01, *** *p* < 0.001 vs. the DMSO group.

The effects of N2 on cell cycle progression were evaluated using propidium iodide (PI) staining and flow cytometry. As shown in Figure 5A–F, N2 treatment led to a dose- and time-dependent accumulation of cells in the G2/M phase in both HEL (Figure 5A–C) and K562 (Figure 5D–F) cells. This finding suggests that N2 exerts its anti-erythroleukemia effect, at least in part, by inducing G2/M phase arrest. Additionally, N2 treatment was associated with cell enlargement and an increase in polyploidy. Specifically, a high concentration of N2 (8 µM) increased the percentage of polyploid cells in HEL (Figure 6A,B) and K562 (Figure 6C,D) cells after 72 h, with a similar trend observed after 96 h. These data indicate that N2 induces G2/M phase arrest and enhances polyploidy in HEL and K562 cells.

### 3.4. Nootkatone Derivative N2 Activates the PKCδ/MAPK Signaling Pathway and Its Downstream Megakaryocytic Differentiation-Related Transcription Factors

**Figure 7:**
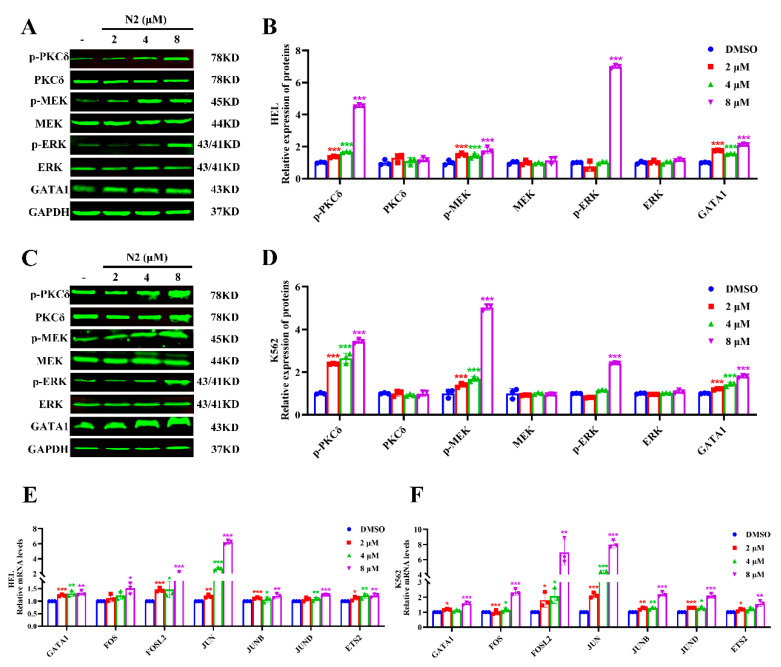
Nootkatone derivative N2 activates the PKCδ/MAPK signaling pathway and its downstream megakaryocytic differentiation-related transcription factors. (**A**–**D**) Upon treating with N2 (2, 4, 8 μM), the expression levels of p-PKCδ, PKCδ, p-MEK, MEK, p-ERK, ERK, and GATA1 detected using Western blotting in HEL (**A**) and K562 (**C**) cells. Densitometry analysis of these proteins in HEL (**B**) and K562 (**D**) cells. (**E**,**F**) Effects of N2 on the mRNA expression levels of seven transcription factors relevant to megakaryocytic differentiation in HEL (**E**) and K562 (**F**) cells. All data are expressed as the mean ± SD. GAPDH was used as loading control. Each experiment was repeated in triplicate. * *p* < 0.05, ** *p* < 0.01, *** *p* < 0.001 vs. the DMSO group.

Previous studies have suggested that activation of the PKCδ/MAPK signaling pathway is essential for megakaryocytic differentiation [25,26,27]. Western blot analysis revealed that N2 treatment enhanced the phosphorylation of PKCδ, MEK, and ERK in a dose-dependent manner, along with an increase in the expression of the megakaryocyte-specific transcription factor GATA1 in both HEL (Figure 7A,B) and K562 (Figure 7C,D) cells. During megakaryocytic differentiation, the biological effect of PKCδ/MAPK signaling in the nucleus is achieved by promoting the transcription of megakaryocytic differentiation related transcription factors including *JUN*, *JUNB*, *FOS*, etc. [28,29]. Consistent with these findings, qRT-PCR analysis showed that N2 upregulated seven transcription factors associated with megakaryocytic differentiation (*GATA1*, *FOS*, *FOSL2*, *JUN*, *JUNB*, *JUND*, and *ETS2*) in a dose-dependent manner in HEL (Figure 7E) and K562 (Figure 7F) cells. These results suggest that N2 promotes megakaryocytic differentiation through activation of the PKCδ/MAPK signaling pathway, leading to the upregulation of key transcription factors.

### 3.5. Nootkatone Derivative N2 Activates the JAK2/STAT3 Signaling Pathway in HEL and K562 Cells

**Figure 8:**
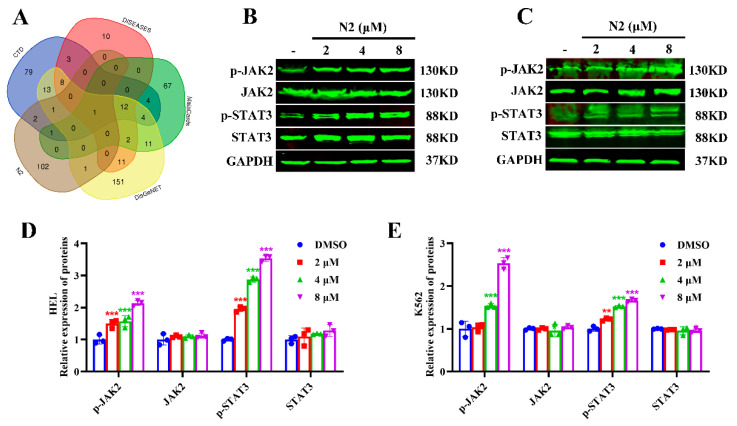
Nootkatone derivative N2 activates the JAK2/STAT3 signaling pathway in HEL and K562 cells. (**A**) JAK2 was identified as a potential target based on the intersection of N2-predicted targets with established targets associated with acute myeloid leukemia. (**B**–**E**) HEL (**B**) and K562 (**C**) cells were treated with N2 at the indicated doses. The expression levels of p-JAK2, JAK2, p-STAT3, and STAT3 were analyzed by Western blotting. Densitometry analysis of p-JAK2, JAK2, p-STAT3, and STAT3 in HEL (**D**) and K562 (**E**) cells. All data are expressed as the mean ± SD, with GAPDH serving as the loading control. Each experiment was repeated in triplicate. ** *p* < 0.01, *** *p* < 0.001 vs. the DMSO group.

To further explore the potential targets and upstream signaling pathways of N2, the SwissTargetPrediction database was used, identifying a total of 108 predicted targets (Table S4). JAK2 was identified as a potential target based on the intersection of N2-predicted targets with known targets of acute myeloid leukemia (AML) (Figure 8A, Tables S5–S8). Given the known involvement of JAK2/STAT3 signaling in megakaryocytic differentiation [30,31], we hypothesized that N2 might activate this pathway. Western blot analysis confirmed that N2 significantly upregulated the phosphorylation of JAK2 and STAT3 in HEL (Figure 8B,D) and K562 (Figure 8C,E) cells. These findings suggest that N2 may promote megakaryocytic differentiation by activating the JAK2/STAT3 signaling pathway.

### 3.6. Nootkatone Derivative N2 Promotes Megakaryocytic Differentiation via Activation of the JAK2/STAT3 and PKCδ/MAPK Signaling Pathways

**Figure 9:**
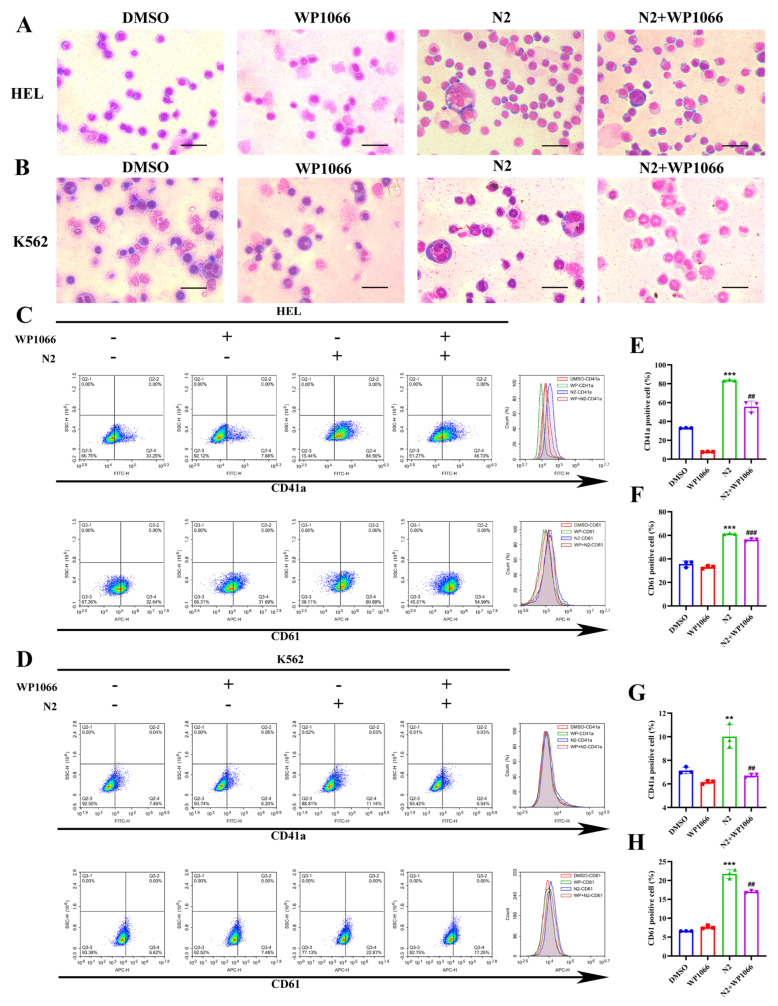
Nootkatone derivative N2 promotes megakaryocytic differentiation via activation of the JAK2/STAT3 signaling pathways. (**A**,**B**) JAK2/STAT3 pathway specific inhibitor WP1066 treatment inhibited N2-induced increases in cell size, multinucleation in HEL and K562 cells via Wright–Giemsa staining. Magnification: ×400. Scale bar: 50 µm. (**C**,**D**) HEL and K562 cells were treated with N2 (8 μM) either alone or in combination with JAK2/STAT3 inhibitor, WP1066 (1 μM), and the expression levels of CD41a and CD61 analyzed using flow cytometry. (**E**–**H**) Quantification of the percentage of CD41a^+^ cells and CD61^+^ cells in HEL (**E**,**F**) and K562 (**G**,**H**) cells. Data are expressed as the mean ± SD. Each experiment was repeated in triplicate. ** *p* < 0.01, *** *p* < 0.001 vs. the DMSO group. ^##^ *p* < 0.01, ^###^ *p* < 0.001 vs. N2 group.

**Figure 10:**
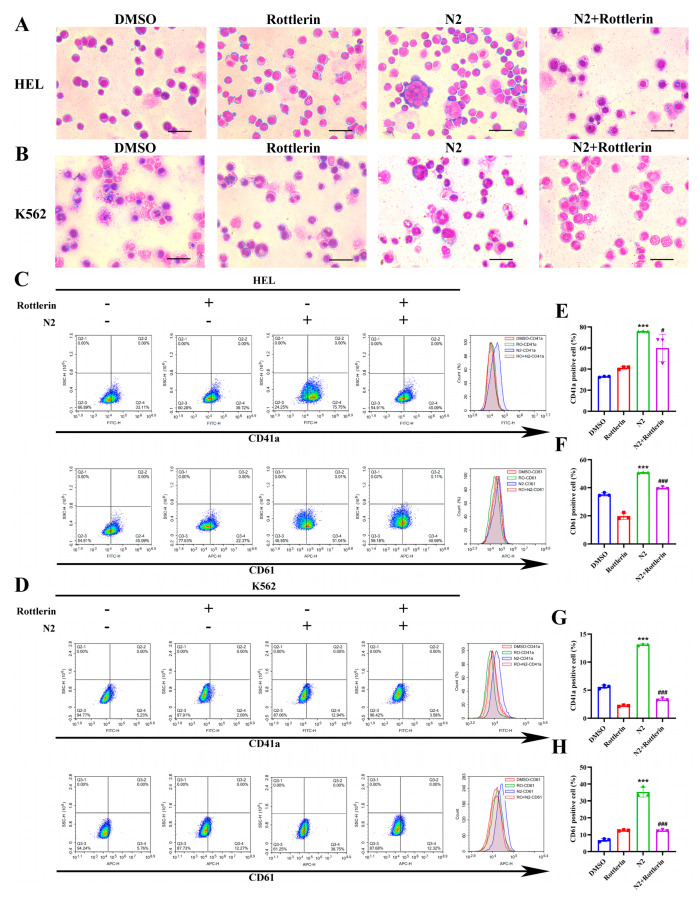
Nootkatone derivative N2 promotes megakaryocytic differentiation via activation of the PKCδ/MAPK signaling pathways. (**A**,**B**) Treatment of HEL and K562 cells with N2 at a concentration of 8 μM, both independently and in conjunction with the PKCδ-specific inhibitor Rottlerin (1 μM). The cells were subjected to Wright–Giemsa staining for morphological assessment. Magnification: ×400. Scale bar: 50 µm. (**C**,**D**) HEL and K562 cells were treated with N2 (8 μM) in the absence or presence of Rottlerin (1 μM), and expression of CD41a and CD61 analyzed using flow cytometry. (**E**–**H**) Quantification of the percentage of CD41a^+^ cells and CD61^+^ cells in HEL (**E**,**F**) and K562 (**G**,**H**) cells. Data are presented as the mean ± SD. Each experiment was repeated in triplicate. *** *p* < 0.001 vs. the DMSO group. ^#^ *p* < 0.05, ^###^ *p* < 0.001 vs. N2 group.

To elucidate the underlying mechanisms of N2-induced differentiation, specific inhibitors of the JAK2/STAT3 (WP1066) and PKCδ (Rottlerin) pathways were employed. As expected, WP1066 treatment inhibited N2-induced increases in cell size, multinucleation (Figure 9A,B), and the expression of CD41a and CD61 in HEL (Figure 9C,E,F) and K562 (Figure 9D,G,H) cells. Similarly, Rottlerin treatment attenuated the N2-induced enhancement of megakaryocytic differentiation (Figure 10A–H). These findings suggest that N2-induced megakaryocytic differentiation is dependent on the activation of both the JAK2/STAT3 and PKCδ/MAPK signaling pathways.

### 3.7. Crosstalk Between JAK2/STAT3 and PKCδ/MAPK Signaling Pathways in Nootkatone Derivative N2-Induced Megakaryocytic Differentiation

**Figure 11:**
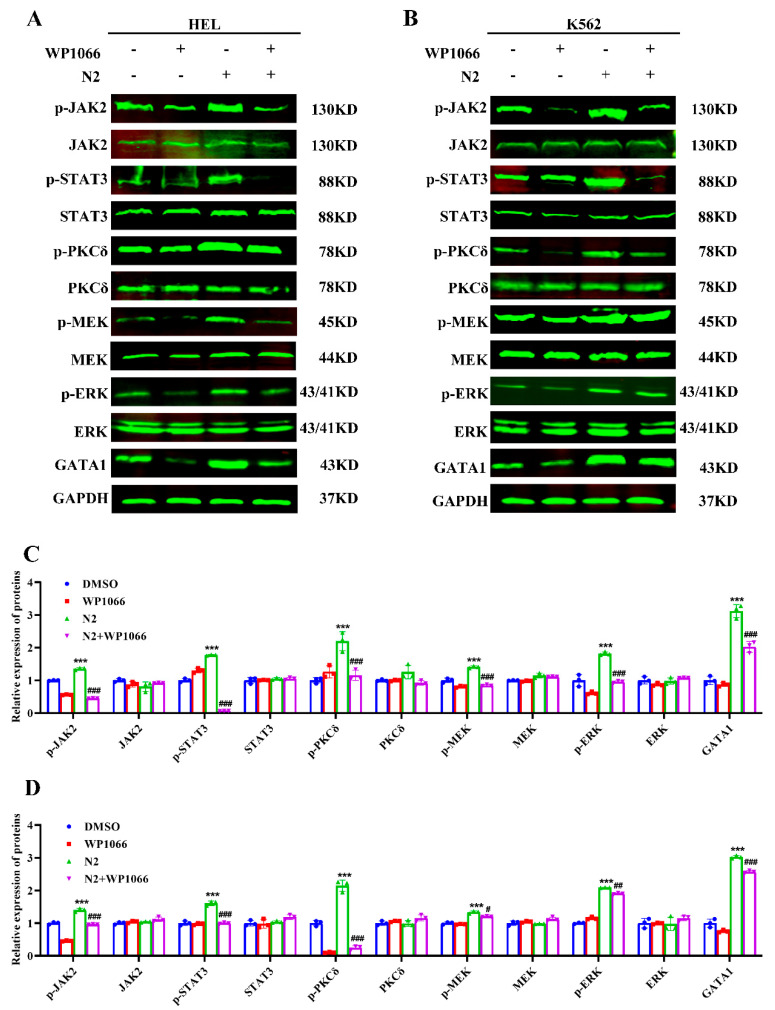
WP1066 treatment significantly reduced N2-induced phosphorylation of JAK2, STAT3, PKCδ, MEK, and ERK, as well as the expression of GATA1. (**A**,**B**) HEL and K562 cells were treated with N2 (8 μM) and/or WP1066 (1 μM), and the protein expression levels of p-JAK2, JAK2, p-STAT3, STAT3, p-PKCδ, PKCδ, p-MEK, MEK, p-ERK, ERK, and GATA1 were detected using Western blotting. (**C**,**D**) Densitometry analysis of these proteins in HEL (**C**) and K562 (**D**) cells. Data are presented as the mean ± SD. GAPDH was used as loading control. Each experiment was repeated in triplicate. *** *p* < 0.001 vs. the DMSO group. ^#^ *p* < 0.05, ^##^ *p* < 0.01, ^###^ *p* < 0.001 vs. N2 group.

**Figure 12:**
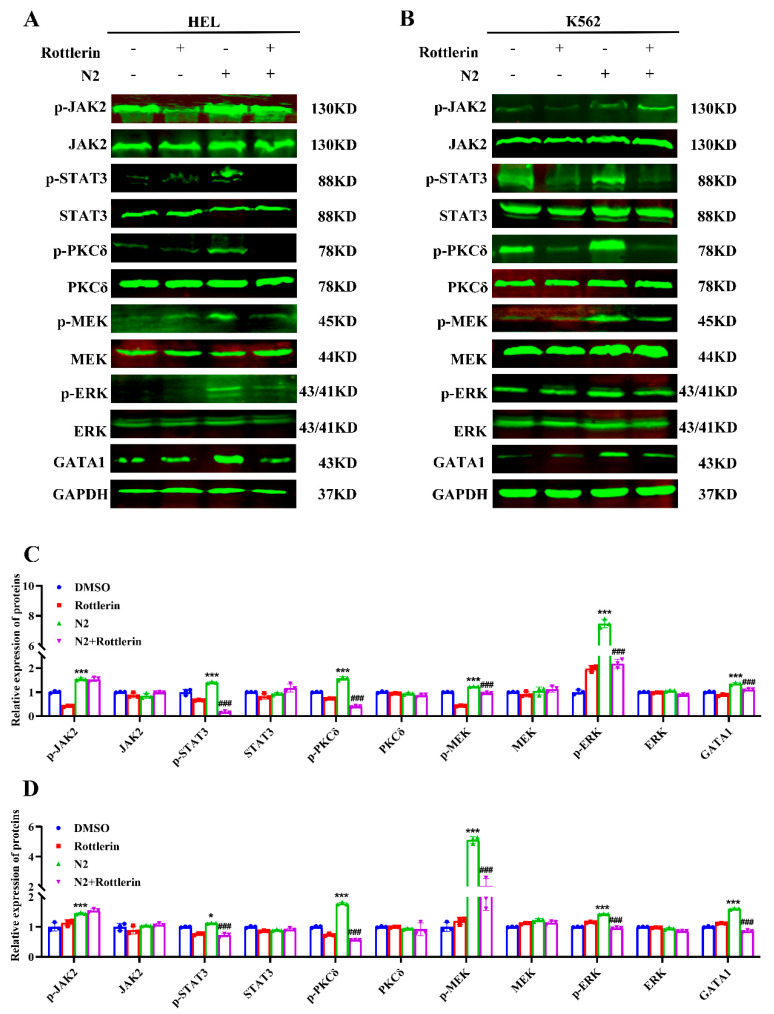
Rottlerin significantly diminished N2-induced PKCδ, STAT3, MEK and ERK phosphorylation. (**A**,**B**) HEL and K562 cells were treated with N2 (8 μM) in the absence or presence of Rottlerin (1 μM), and the proteins expression of p-JAK2, JAK2, p-STAT3, STAT3, p-PKCδ, PKCδ, p-MEK, MEK, p-ERK, ERK and GATA1 were detected using Western blotting. (**C**,**D**) Densitometry analysis of these proteins in HEL (**C**) and K562 (**D**) cells. Data are presented as the mean ± SD. GAPDH was used as loading control. Each experiment was repeated in triplicate. * *p* < 0.05, *** *p* < 0.001 vs. the DMSO group. ^###^ *p* < 0.001 vs. N2 group.

JAK2/STAT3 and PKCδ/MAPK signaling pathways are crucial for megakaryocytic differentiation, with crosstalk between them previously demonstrated [32]. To determine whether N2 activates this crosstalk, the JAK2 inhibitor WP1066 and the PKCδ inhibitor Rottlerin were used. WP1066 treatment significantly reduced N2-induced phosphorylation of JAK2, STAT3, PKCδ, MEK, and ERK, as well as the expression of GATA1 in HEL (Figure 11A,C) and K562 (Figure 11B,D) cells. Then, we examined the effects of Rottlerin, a PKCδ inhibitor, on N2-induced megakaryocytic differentiation. We found that Rottlerin had no effects on JAK2 in N2-induced megakaryocytic differentiation (Figure 12A–D), indicating that PKC functions downstream of JAK2 in the JAK2/STAT3 pathway. However, Rottlerin significantly diminished N2-induced PKCδ, STAT3, MEK, and ERK phosphorylation (Figure 12A–D), indicating that PKCδ may be a downstream target of JAK2 in megakaryocytic differentiation, and N2-induced differentiation might also involve JAK2 signaling independently of PKCδ. These results suggest that N2 promotes megakaryocytic differentiation through the crosstalk between JAK2/STAT3 and PKCδ/MAPK signaling pathways.

### 3.8. Nootkatone Derivative N2 Binds to JAK2

**Figure 13:**
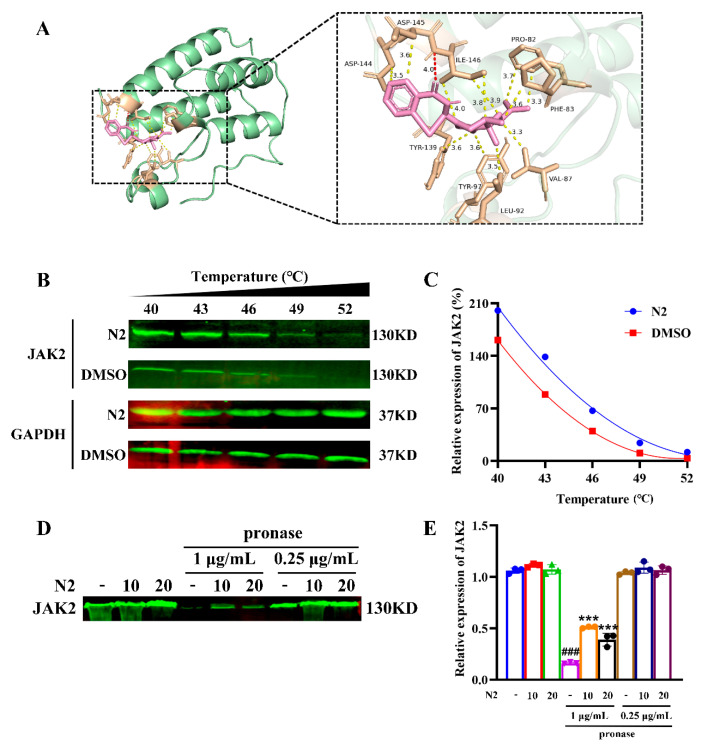
Nootkatone derivative N2 binds to JAK2. (**A**) Molecular docking of N2 and JAK2 was conducted using AutoDock Vina 1.1.2. (**B**) CETSA was performed to assess the binding interactions between N2 and JAK2. (**C**) The stability of the JAK2 protein across varying temperatures was quantified using Western blotting analysis. (**D**) The DARTS experiments confirmed the binding of N2 to the JAK2 protein. (**E**) Densitometry analysis of JAK2. Data are presented as the mean ± SD. Each experiment was repeated in triplicate. ^###^ *p* < 0.001 vs. lysates group, *** *p* < 0.001 vs. pronase alone group.

Through network pharmacology analysis (Figure 8) and preliminary experimental verification (Figure 9, Figure 10, Figure 11 and Figure 12), we speculated that JAK2 is a potential target of N2. First, we performed molecular docking between JAK2 and N2. We found that N2 can form hydrogen bonds with ILE-146 in JAK2 and hydrophobic interactions with PRO-82, PHE-83, VAL-87, LEU-92, TYR-97, TYR-139, ASP-144, ASP-145, and ILE-146 (Figure 13A). The results showed that the binding affinity score of N2 to JAK2 was -8.8 kcal/mol, indicating a strong docking between N2 and JAK2. Cellular thermal shift assay (CETSA) results further demonstrated increased thermal stability of JAK2 in N2-treated cells (Figure 13B,C), indicating direct interaction. Drug affinity responsive target stability (DARTS) analysis also confirmed that N2 protected JAK2 from proteolytic degradation (Figure 13D,E), supporting the hypothesis that N2 binds to JAK2.

### 3.9. Nootkatone Derivative N2-Mediated Megakaryocytic Differentiation in Erythroleukemia Cells Is JAK2-Dependent

**Figure 14:**
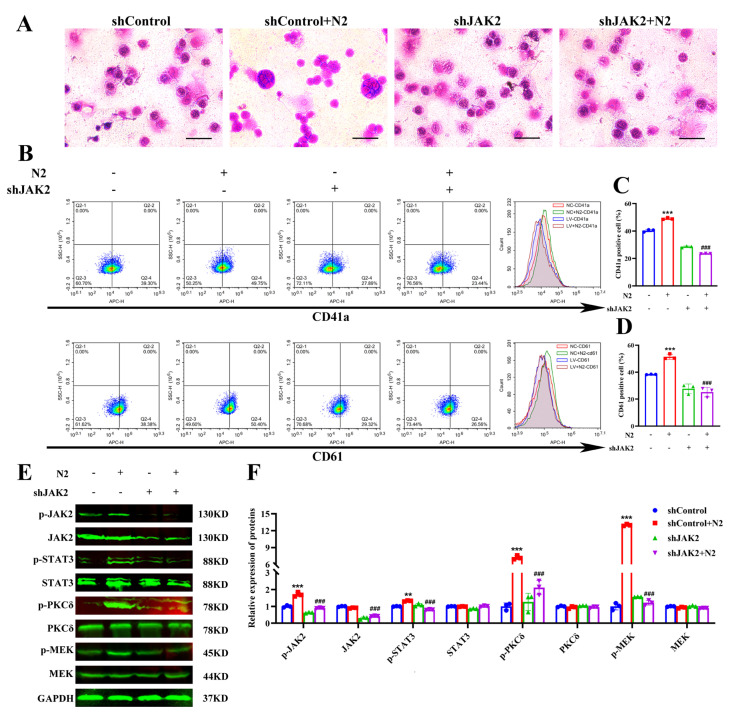
Nootkatone derivative N2-mediated megakaryocytic differentiation in erythroleukemia cells is JAK2-dependent. (**A**) Morphological analysis was conducted using Wright–Giemsa staining on LV-NC and LV-sh-JAK2 HEL cells exposed to 8 μM N2. Magnification: ×400. Scale bar: 50 µm. (**B**) Flow cytometry was employed to assess the expression levels of CD41a and CD61 in LV-NC and LV-sh-JAK2 HEL cells treated with 8 μM N2. (**C**,**D**) Quantification of the percentage of CD41a^+^ and CD61^+^ cells. (**E**) The expression of p-JAK2, JAK2, p-PKCδ, PKCδ, p-STAT3, STAT3, p-MEK, and MEK in LV-NC and LV-sh-JAK2 HEL cells treated with 8 μM N2 were measured by Western blotting. (**F**) Densitometry analysis of these proteins. All data are presented as the mean ± SD. Each experiment was repeated in triplicate. ** *p* < 0.01, *** *p* < 0.001 vs. LV-NC/DMSO group. ^###^ *p* < 0.001 vs. LV-NC/N2-8 µM group.

To further explore the role of JAK2 in N2-induced megakaryocytic differentiation, JAK2 expression was knocked down in HEL cells via lentiviral infection with either LV-sh-JAK2 or LV-NC. The knockdown efficiency was confirmed by fluorescence microscopy (Figure S1A) and Western blot analysis (Figure S1B,C). As shown in Figure 14A, the knockdown of JAK2 in HEL cells significantly attenuated the N2-induced increases in cell size and nuclear number, as observed via Wright–Giemsa staining. Additionally, the upregulation of CD41a and CD61 levels in response to N2 treatment was significantly reduced in JAK2-knockdown cells (Figure 14B–D). Consistently, Western blot analysis revealed that JAK2 knockdown markedly suppressed the N2-mediated phosphorylation of JAK2, PKCδ, STAT3, and MEK in HEL cells (Figure 14E,F). These findings collectively demonstrate that N2 promotes megakaryocytic differentiation in erythroleukemia cells through the activation of JAK2.

### 3.10. Nootkatone Derivative N2 Accelerates Megakaryocytic Differentiation and Suppresses Erythroleukemia In Vivo

**Figure 15:**
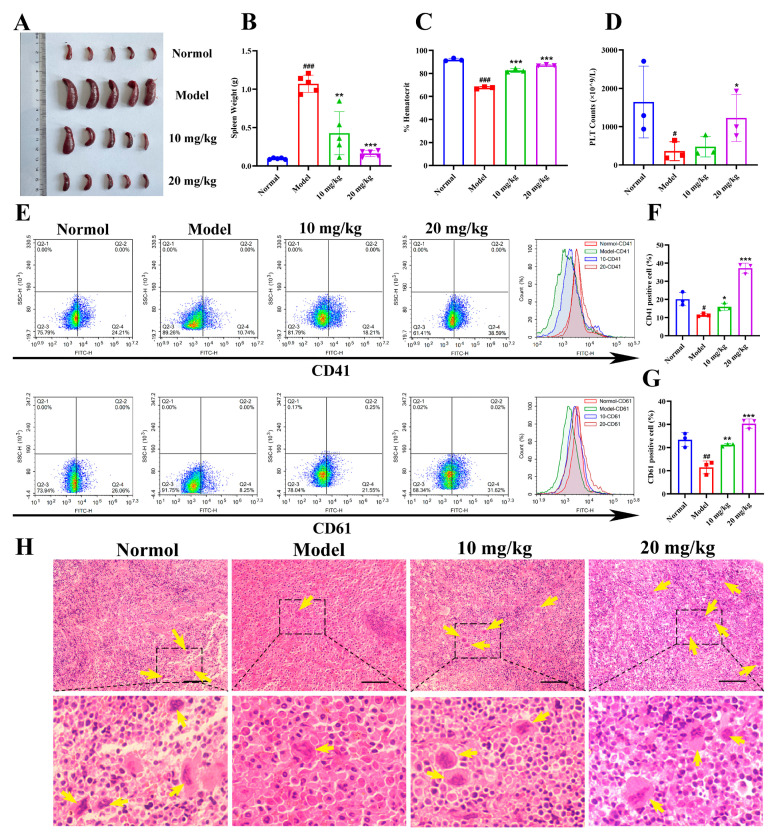
Nootkatone derivative N2 accelerates megakaryocytic differentiation and suppresses erythroleukemia in vivo. (**A**) Spleen size of mice in different experimental groups. (**B**) Spleen weight presented as mean ± SD (*n* = 5). (**C**) Hematocrit values. (**D**) Platelet counts. (**E**) Flow cytometric analysis of CD41 and CD61 expression of spleen of each groups. (**F**,**G**) The histogram represents the percentage of CD41^+^ and CD61^+^ cells of spleen in each groups. (**H**) Representative H&E stained images of spleen from each groups. The yellow arrow represents megakaryocytes. Magnification: ×400. Scale bar: 50 µm. Data represent the mean ± SD (*n* = 3). * *p* < 0.05, ** *p* < 0.01, *** *p* < 0.001 vs. model group. ^#^ *p* < 0.05, ^##^ *p* < 0.01, ^###^ *p* < 0.001 vs. normal group.

**Figure 16:**
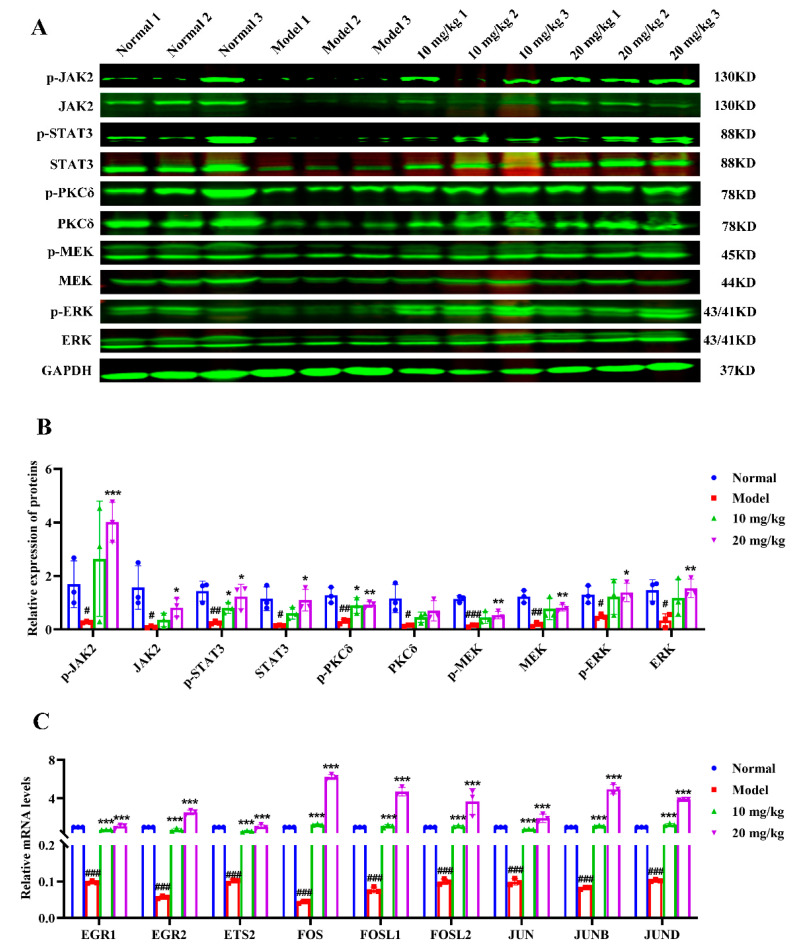
Nootkatone derivative N2 activates JAK2/STAT3 and PKCδ/MAPK signaling pathways. (**A**) The expression of p-JAK2, JAK2, p-PKCδ, PKCδ, p-STAT3, STAT3, p-MEK, MEK, p-ERK, and ERK of spleen tissue from each groups were measured by Western blotting. (**B**) Densitometry analysis of these proteins. (**C**) The impact of N2 on the mRNA expression of nine transcription factors related to megakaryocytic differentiation in spleen tissue. Data are expressed as the mean ± SD (*n* = 3). * *p* < 0.05, ** *p* < 0.01, *** *p* < 0.001 vs. model group. ^#^ *p* < 0.05, ^##^ *p* < 0.01, ^###^ *p* < 0.001 vs. normal group.

To evaluate the in vivo effects of N2, an allograft erythroleukemia model was established by injecting murine erythroleukemia CB3 cells into BALB/c mice via tail veins [33]. As shown in the results, the spleen size and weight in the model group were significantly larger than those in the control group. Treatment with N2 (10 mg/kg and 20 mg/kg) significantly reduced spleen size and weight (Figure 15A,B). Mice treated with N2 exhibited increased hematocrit levels (Figure 15C) and platelet counts (Figure 15D) compared to the model group. Furthermore, flow cytometric analysis revealed a significant increase in the percentage of CD41- and CD61-positive spleen cells in the N2-treated group (Figure 15E–G). H&E staining demonstrated a marked increase in the number of megakaryocytes in the spleens of N2-treated mice (Figure 15H), indicating that N2 promotes megakaryocytic differentiation in vivo. In line with the in vitro findings, Western blot analysis confirmed that N2 treatment upregulated the phosphorylation of JAK2, STAT3, PKCδ, MEK, and ERK in spleen tissue (Figure 16A,B). Additionally, qRT-PCR analysis showed that N2 significantly upregulated the expression of transcription factors associated with megakaryocytic differentiation, including *EGR1*, *EGR2*, *ETS2*, *FOS*, *FOSL1*, *FOSL2*, *JUN*, *JUNB*, and *JUND* (Figure 16C). Importantly, after N2 treatment no pathological changes were observed in major organs such as the lungs, heart, liver, or kidneys (Figure S2A), and there was no apparent impact on the body weight of mice across all groups (Figure S2B). Additionally, the levels of liver and kidney function markers, including TBIL, CRE, BUN, GOT, and GPT, remained unchanged after N2 treatment (Figure S2C–G), suggesting that N2 exhibits no significant toxicity in vivo.

## 4. Discussion

Nootkatone is a sesquiterpene compound derived from the ketone family of valencene. It is found in higher concentrations in grapefruit compared to other citrus species and is recognized for its distinct grapefruit flavor. Due to its non-toxic nature and safety in humans and other mammals, nootkatone has been classified as “generally recognized as safe” (GRAS) by the U.S. Food and Drug Administration (FDA) [34]. This designation allows for its widespread use as a flavoring agent and additive in beverages, food, and essential oils [35,36]. Additionally, nootkatone exhibits a broad range of biological activities. For instance, it serves as an effective biopesticide to protect against insect bites [37,38], and in the pharmaceutical industry, it is a key component of grapefruit oil used in aromatherapy. Nootkatone has been shown to improve body image in postmenopausal women, alleviate stress, and boost immunity [39,40]. Given these promising health benefits, nootkatone not only has functional applications on its own but can also be used as a starting material for the synthesis of value-added derivatives with enhanced properties.

Recent studies have revealed that nootkatone derivatives containing amine groups exhibit stronger insecticidal activity than the parent compound [41]. Furthermore, a series of fused-thiazole derivatives of nootkatone have been synthesized, some of which have demonstrated potent antimicrobial activity against *Staphylococcus aureus* and *Enterococcus faecium* [42]. Other research has shown that N-acyl-2-aminothiazole-based derivatives of nootkatone possess potent α-glucosidase inhibitory properties, suggesting their potential as antidiabetic agents [43]. Additionally, Alam et al. synthesized 50 novel compounds based on nootkatone-derived epoxyketones, and several ethisterone-based fused-thiazole compounds displayed significant anticancer activity [44]. Our research group previously synthesized nootkatone derivatives by introducing oxime ester and acylhydrazone structures, and one derivative, N17, exhibited potent inhibitory activity against *Phomopsis* sp. [21]. In the present study, we investigated the effects of nootkatone and its derivatives on erythroleukemia cells and found that the derivative N2 demonstrated notable anti-erythroleukemia activity by inducing megakaryocytic differentiation in both in vitro and in vivo models.

Platelets are specialized cells that play a crucial role in hemostasis and vascular repair. They are derived from megakaryocytes, which release platelets by extending long, branching proplatelets into the sinusoidal blood vessels [45]. Hematopoietic stem cells (HSCs) give rise to megakaryocytes through a series of controlled differentiation and maturation processes involving multiple progenitor stages, including multipotent progenitor cells, common myeloid progenitors, and megakaryocyte-erythroid progenitors (MEP) [46]. Megakaryocytic differentiation is characterized by significant morphological and functional changes, including an increase in cell volume, polyploidization, and the enhanced expression of surface markers such as CD41, CD42, and CD61 [47]. Since acute erythroleukemia (AEL) arises from the erythroid and megakaryocytic lineages derived from MEP, it retains the potential for megakaryocytic differentiation [4]. Previous studies have suggested that nootkatone may influence tumor cell stemness [20], leading us to hypothesize that it might similarly affect leukemia stemness and differentiation. In our study, we found that nootkatone derivative N2 induced megakaryocytic differentiation, as evidenced by increased cell size, polyploid nuclei, and enhanced expression of CD41 and CD61 in HEL and K562 cells. In vivo, N2 administration increased platelet counts, while spleen histology and flow cytometry confirmed an elevated number of splenic megakaryocytes and promoted megakaryocytic differentiation.

Megakaryocytic differentiation is tightly regulated by various cytokines, particularly thrombopoietin (TPO). TPO is a critical enhancer of megakaryocytic differentiation and platelet production through its interaction with the thrombopoietin receptor (c-MPL) [48]. Activation of the TPO/c-MPL signaling axis triggers multiple pathways, including JAK2 [49], STAT3/STAT5 [50], PKC/MAPK/ERK [51,52], and PI3K/AKT [53]. A previous study demonstrated that the JAK2/STAT3 and JAK2/PKCδ/STAT3 pathways cooperatively regulate megakaryocytic differentiation [32], suggesting a crosstalk between downstream signaling cascades of the TPO/c-MPL pathway. Besides TPO, other cytokines, such as IL-3, IL-6, IL-9, IL-11, and stem cell factor (SCF), also play roles in stimulating megakaryocyte production. SCF, for example, promotes cell proliferation during early megakaryocyte differentiation, while IL-3 synergizes with TPO to enhance differentiation [54]. Transcription factors such as *GATA1*, *GATA2*, *FOS*, *JUN*, and *RUNX1* play pivotal roles in regulating MEP lineage determination during megakaryocytic differentiation [55]. Moreover, emerging evidence suggests that epigenetic mechanisms, including DNA methylation, histone modifications, and non-coding RNA regulation, are key regulators of megakaryocyte development and platelet production [56]. Despite these advancements, the mechanisms governing megakaryocytic differentiation remain incompletely understood. The use of differentiation inducers as molecular probes may help elucidate novel pathways involved in this process.

A hallmark of acute myeloid leukemia (AML) is the impairment of differentiation. Therapeutic agents that induce differentiation are therefore of great clinical value. In the case of acute promyelocytic leukemia (APL), a unique subtype of AML, treatment with all-trans retinoic acid (ATRA) and arsenic trioxide (ATO) induces the differentiation of leukemic cells into normal myeloid cells, leading to significant therapeutic outcomes and a 5-year survival rate exceeding 95% [57]. However, due to the high heterogeneity of AML, differentiation therapy is less effective in other subtypes of the disease. Several small molecules have been identified to promote erythroid and megakaryocytic differentiation in AEL. For instance, hirsutine (HS), an alkaloid isolated from *Uncaria* and *Mitragyna* genera, induces megakaryocytic differentiation in K562 and Meg01 cells by activating the MEK/ERK pathway and upregulating transcription factors such as *FOG1* and *GATA1* [58]. Proanthocyanidin A1, derived from peanut skin, was the first compound to be identified as an inducer of megakaryocytic differentiation through the JAK2/STAT3 pathway in both in vitro and in vivo studies [59]. Another example is 3,3’,4’-trimethylellagic acid (TMEA), a polyphenol found in *Sanguisorba officinalis* L., which promotes megakaryocytic differentiation and platelet production in HEL cells via the PI3K/Akt/mTOR/P70S6K/GATA1/NF-E2 pathway [60]. Interestingly, most reported small molecules that induce megakaryocytic differentiation act through independent signaling pathways. Unlike previous studies, our findings substantiated that nootkatone derivative N2 can induce megakaryocytic differentiation by binding and targeting JAK2. This interaction activates a crosstalk between the JAK2/STAT3 and PKCδ/MAPK pathways, leading to the upregulation of megakaryocytic differentiation-related transcription factors, including *GATA1*, *FOS*, *FOSL2*, *JUN, JUNB*, *JUND*, and *ETS2*. Ultimately, this mechanism contributes to the anti-erythroleukemia effects observed with N2 treatment.

In this study, we investigated the preventative effects of N2 and its role in early disease intervention. While our findings highlight the promising therapeutic potential of N2, further research is needed to evaluate its efficacy in later-stage leukemia models, which would more closely mimic clinical scenarios. We observed that long-term administration of N2 did not adversely affect major organs, including the lungs, heart, liver, and kidneys. However, we did not assess the long-term repopulation potential of hematopoietic stem cells (HSCs) through secondary transplantation assays. Addressing this limitation is critical for a comprehensive safety profile. Future studies should include secondary transplantation experiments to assess the impact of N2 on HSC function and monitor hematopoietic recovery over extended periods.

Despite N2’s demonstrated efficacy in anti-leukemia activity, long-term use may lead to the development of resistance in leukemia cells, a common challenge in targeted therapies. This resistance could arise from several mechanisms. First, leukemia cells may acquire mutations in molecular targets or signaling pathways affected by N2, diminishing their sensitivity to the compound. Similar resistance mechanisms have been observed with tyrosine kinase inhibitors (TKIs) [61], where mutations in drug-binding sites compromise therapeutic efficacy. Second, alterations in the epigenetic landscape, such as changes in chromatin accessibility or histone modifications, may enable leukemia cells to adapt and survive in the presence of N2. This phenomenon has been reported in resistance to therapies targeting differentiation pathways [62]. Additionally, leukemia stem cells (LSCs), which are often quiescent and inherently resistant to many treatments, may evade the effects of N2. These cells could potentially repopulate the leukemia and drive disease relapse over time [63]. Understanding these mechanisms is critical in overcoming resistance and improving therapeutic outcomes. In light of these possibilities, further studies are needed to evaluate whether and how leukemia cells might develop resistance to N2. For example, long-term in vitro exposure models and in vivo relapse studies could be employed to identify potential resistance mechanisms.

## 5. Conclusions

**Figure 17:**
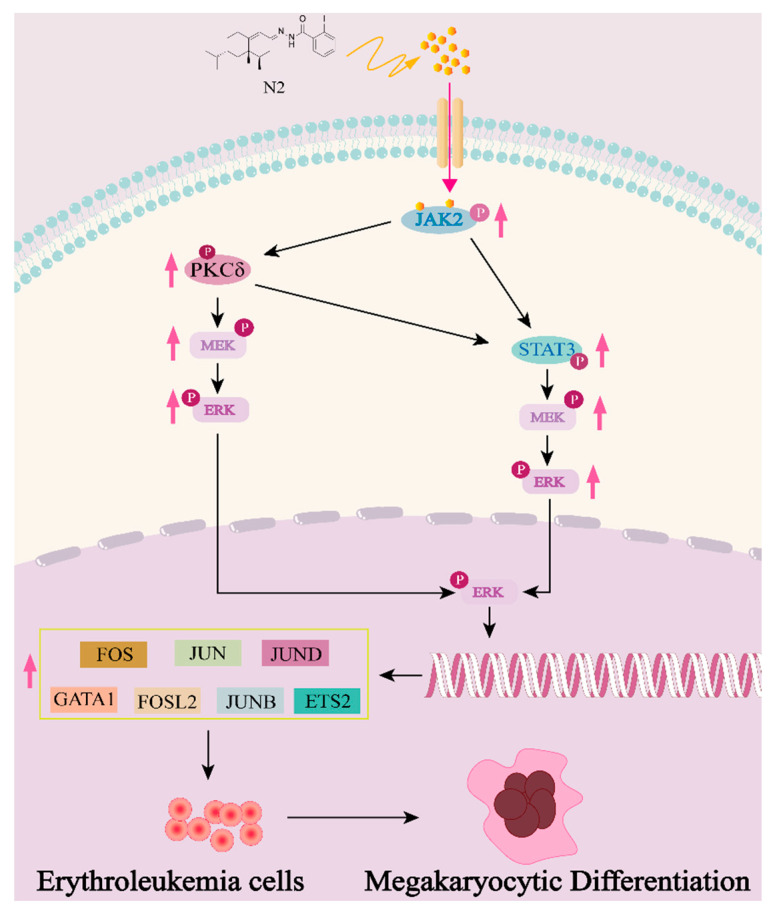
Illustration of the role and mechanism of nootkatone derivative N2 in promoting megakaryocytic differentiation in erythroleukemia. The red arrow indicates up-regulation.

In summary, N2 represents a potent differentiation therapy candidate for erythroleukemia, uniquely targeting JAK2 and activating downstream JAK2/STAT3, JAK2/PKCδ/STAT3 and JAK2/PKCδ/MAPK pathways to facilitate megakaryocytic differentiation (Figure 17). These findings support continued investigation into nootkatone derivatives and natural compounds as viable options for targeted leukemia therapies.

## Data Availability

The data presented in this study are available on request from the corresponding authors.

## Supplementary Materials

The following supporting information can be downloaded at: https://www.mdpi.com/article/10.3390/cells14010010/s1, Figure S1: The knockdown efficiency was confirmed by fluorescence microscopy and Western blotting analysis. (**A**) Fluorescent images of stable cell lines. Magnification: ×200. Scale bar: 100 µm. (**B**) The efficiency of JAK2 knockdown was further corroborated by Western blotting. (**C**) Densitometry analysis of JAK2. Data are presented as the mean ± SD. Each experiment was repeated in triplicate. *** *p* < 0.001 vs. shControl group; Figure S2: N2 exhibits no significant toxicity in vivo. (**A**) Representative images of H&E staining of lungs, heart, liver, or kidneys in each groups. Magnification: ×400. Scale bar: 50 µm. (**B**) Body weight expressed as mean ± SD (*n* = 6). (**C**–**G**) The levels of liver and kidney function markers, in-cluding TBIL (**C**), CRE (**D**), BUN (**E**), GOT (**F**), and GPT (**G**). Data are expressed as the mean ± SD (*n* = 3); Table S1: The sequences of primers used in this study; Table S2: The primary and secondary antibodies used in this study; Table S3: IC_50_ of nootkatone and its derivatives; Table S4: Results of predictied targets through SwissTargetPrediction; Table S5: The targets of AML from CTD; Table S6: The targets of AML from DISEASES; Table S7: The targets of AML from MalaCards; Table S8: The targets of AML from DisGeNET.

## Abbreviations

AML, acute myeloid leukemia; AEL, acute erythroleukemia; MEP, megakaryocyte-erythrocyte progenitor; ATCC, American type culture collection; RPMI, Roswell Park Memorial Institute; DMEM, Dulbecco’s modified eagle’s medium; FBS, fetal bovine serum; OD, optical density; PI, propidium iodide; qRT-PCR, quantitative real-time PCR; JAK2, Janus kinase 2; p-JAK2, phosphor-Janus kinase 2; STAT3, signal transducer and activator of transcription 3; p-STAT3, phosphor-signal transducer and activator of transcription 3; PKCδ, protein kinase C-delta; p-PKCδ, phosphor-protein kinase C-delta; MEK, mitogen-activated extracellular signal-regulated kinase; p-MEK, phosphor-mitogen-activated extracellular signal-regulated kinase; ERK, extracellular regulated protein kinases; p-ERK, phosphor-extracellular regulated protein kinases; GAPDH, glyceraldehyde-3-phosphate dehydrogenase; PBS, phosphate buffer saline; PDB, Protein Data Bank; CETSA, cellular thermal shift assay; DMSO, dimethyl sulfoxide; DARTS, drug affinity responsive target stability; H&E, hematoxylin and eosin; TBIL, total bilirubin; CRE, creatinine; BUN, blood urea nitrogen; GOT, glutamic oxaloacetic transaminase; GPT, glutamic pyruvic transaminase; SD, standard deviation; ANOVA, one-way analysis of variance; IC_50_, half-maximal inhibitory concentration; MAPK, mitogen-activated protein kinase; RIPA, radioimmunoprecipitation assay; GRAS, generally recognized as safe; FDA, Food and Drug Administration; TPO, thrombopoietin; c-MPL, thrombopoietin receptor; SCF, stem cell factor; APL, acute promyelocytic leukemia; ATRA, all-trans retinoic acid; ATO, arsenic trioxide; HS, hirsutine; TMEA, 3,3’,4’-trimethylellagic acid; FSC, forward scatter; SSC, side scatter; HSCs, hematopoietic stem cells; TKIs, tyrosine kinase inhibitors; LSCs, leukemia stem cells.
