# Supplementary material for: Nootkatone Derivative Nootkatone-(E)-2-iodobenzoyl hydrazone Promotes Megakaryocytic Differentiation in Erythroleukemia by Targeting JAK2 and Enhancing JAK2/STAT3 and PKCδ/MAPK Crosstalk
Source: Cells. 2024 Dec 26;14(1):10. doi: 10.3390/cells14010010 (PMC11720125; doi:10.3390/cells14010010)
Supplement: Supplementary file 1 [file cells-14-00010-s001.zip › Revised-Table S1.pdf]

Table S1 The sequences of primers used in this study.

Genes	Primer sequence
H-GAPDH	Forward primer: 5′- GTCTCCTCTGACTTCAACAGCG -3
	Reverse primer: 5′- ACCACCCTGTTGCTGTAGCCAA -3′
H-GATA1	Forward primer: 5′- TGGTGGCTTTATGGTGGTG -3′
	Reverse primer: 5′- CCTTGGTAGAGATGGGCAGT -3′
H-FOSL2	Forward primer: 5′- CAGGTTGCTGTCCCTCACAT -3′
	Reverse primer: 5′- CTCGGGGCTAATCTTGCACA -3′
H-JUN	Forward primer: 5′- CCTTGAAAGCTCAGAACTCGGAG -3′
	Reverse primer: 5′- TGCTGCGTTAGCATGAGTTGGC -3′
H-JUNB	Forward primer: 5′- CGATCTGCACAAGATGAACCACG -3′
	Reverse primer: 5′- CTGCTGAGGTTGGTGTAAACGG -3′
H-FOS	Forward primer: 5′- GCCTCTCTTACTACCACTCACC -3′
	Reverse primer: 5′- AGATGGCAGTGACCGTGGGAAT -3′
H-JUND	Forward primer: 5′- ATCGACATGGACACGCAGGAGC -3′
	Reverse primer: 5′- CTCCGTGTTCTGACTCTTGAGG -3′
H-ETS2	Forward primer: 5′- ACTCCGCCAACTGTGAATTGCC -3′
	Reverse primer: 5′- CCACTGGCATACCTGTTGCTCA -3′
M-GAPDH	Forward primer: 5′- AGGTCGGTGTGAACGGATTTG -3′
	Reverse primer: 5′- TGTAGACCATGTAGTTGAGGTCA -3′
M-JUN	Forward primer: 5′- CAGTCCAGCAATGGGCACATCA -3′
	Reverse primer: 5′- GGAAGCGTGTTCTGGCTATGCA -3′
M-JUNB	Forward primer: 5′- GACCTGCACAAGATGAACCACG -3′
	Reverse primer: 5′- ACTGCTGAGGTTGGTGTAGACG -3′
M-JUND	Forward primer: 5′- ACCTGCACAAGCAAAGCCAGCT -3′
	Reverse primer: 5′- CGAAACTGCTCAGGTTGGCGTA -3′
M-FOS	Forward primer: 5′- GGGAATGGTGAAGACCGTGTCA -3′
	Reverse primer: 5′- GCAGCCATCTTATTCCGTTCCC -3′
M-EGR2	Forward primer: 5′- CCTTTGACCAGATGAACGGAGTG -3′

	Reverse primer: 5′- CTGGTTTCTAGGTGCAGAGATGG -3′
M-FOSL1	Forward primer: 5′- ATGTACCGAGACTACGGGGAA -3′
	Reverse primer: 5′- CACCATCCAGTGCAGTTCCT -3′
M-FOSL2	Forward primer: 5′- TCCACGCTCACATCCCTACA -3′
	Reverse primer: 5′- CCTCCGGATTCGCTTCT -3′
M-EGR1	Forward primer: 5′- AGCGAACAACCCTATGAGCACC -3′
	Reverse primer: 5′- ATGGGAGGCAACCGAGTCGTTT -3′
M-ETS2	Forward primer: 5′- GTGGCTTCCAAAAGGAGCAACG -3′
	Reverse primer: 5′- TTCACCAGGCTGAACTCGTTGG -3′
