# Supplementary material for: Nootkatone Derivative Nootkatone-(E)-2-iodobenzoyl hydrazone Promotes Megakaryocytic Differentiation in Erythroleukemia by Targeting JAK2 and Enhancing JAK2/STAT3 and PKCδ/MAPK Crosstalk
Source: Cells. 2024 Dec 26;14(1):10. doi: 10.3390/cells14010010 (PMC11720125; doi:10.3390/cells14010010)
Supplement: Supplementary file 1 [file cells-14-00010-s001.zip › Revised-Table S2.pdf]

Table S2 The primary and secondary antibodies used in this study.

Alternative Names	Company	Catalog number	Host	Dilution
Anti-JAK2 (phospho Y1007+Y1008) antibody [E132]	Abcam, Cambridge, UK	AB32101	Rabbit	1:1000
Anti-JAK2 antibody [EPR108(2)]	Abcam, Cambridge, UK	AB108596	Rabbit	1:5000
Anti-STAT3 (phospho Y705) antibody [EP2147Y]	Abcam, Cambridge, UK	AB76315	Rabbit	1:2000
Anti-STAT3 antibody [EPR787Y]	Abcam, Cambridge, UK	AB68153	Rabbit	1:2000
Anti-PKC delta (phospho Y311) antibody [EPR2609Y]	Abcam, Cambridge, UK	AB76181	Rabbit	1:5000
Anti-PKC delta antibody [EPR17075]	Abcam, Cambridge, UK	AB182126	Rabbit	1:5000
Phospho-MEK1/2 (Ser217/221) (41g9)	CST, Danvers, MA, USA	#9154	Rabbit	1:1000
Anti-MEK1/2 Antibody [SR13-07]	HuaBio, Hangzhou, China	ET1602-3	Rabbit	1:1000
Anti-Erk1 (pT202/pY204)+Erk2 (pT185/pY187) Antibody [SC58-01]	HuaBio, Hangzhou, China	ET1610-13	Rabbit	1:2000
Anti-ERK1/2 Antibody [SA43-03]	HuaBio, Hangzhou, China	ET1601-29	Rabbit	1:5000
GATA-1 (D52H6) XP Rabbit mAb	CST, Danvers, MA, USA	#3535	Rabbit	1:1000
Rabbit anti-GAPDH	GOODHERE BIOTECH, Hangzhou, China	AB-P-R001	Rabbit	1:1000
Anti-rabbit IgG (H+L) (DyLight 800 4X PEG Conjugate)	CST, Danvers, MA, USA	#5151	Rabbit	1:30000
