# Supplementary material for: Nootkatone Derivative Nootkatone-(E)-2-iodobenzoyl hydrazone Promotes Megakaryocytic Differentiation in Erythroleukemia by Targeting JAK2 and Enhancing JAK2/STAT3 and PKCδ/MAPK Crosstalk
Source: Cells. 2024 Dec 26;14(1):10. doi: 10.3390/cells14010010 (PMC11720125; doi:10.3390/cells14010010)
Supplement: Supplementary file 1 [file cells-14-00010-s001.zip › Revised-Table S3.pdf]

Table S3 IC_{50} of nootkatone and its derivatives ($\bar{x} \pm s$, n=3).

Compound	Structure	IC_{50} (μM)	
		HEL	K562
Nootkatone	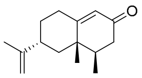	> 20	> 20
N1	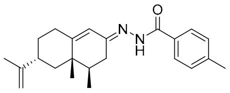	> 20	> 20
N2	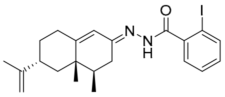	4.58±0.15	6.54±0.27
N3	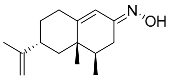	> 20	> 20
N4	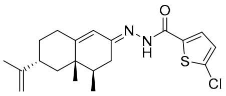	8.57±0.54	> 20
N7	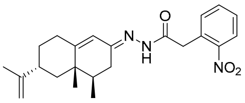	> 20	> 20
N10	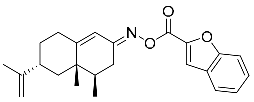	> 20	> 20
N11	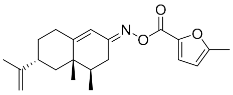	> 20	> 20
N12	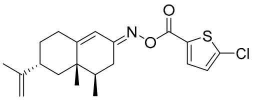	> 20	> 20
N14	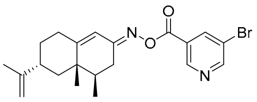	> 20	> 20
N15	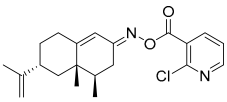	> 20	> 20
N17	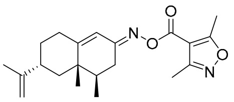	> 20	> 20
N23	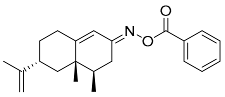	> 20	> 20
N24	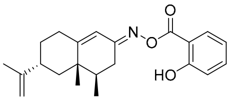	> 20	> 20

N26	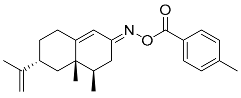	> 20	> 20
N28	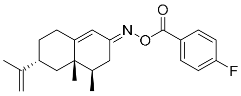	> 20	> 20
N47	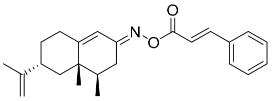	> 20	> 20
N48	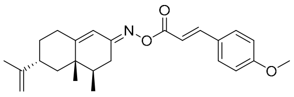	> 20	> 20
N49	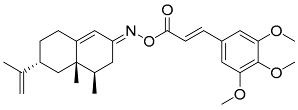	> 20	> 20
N50	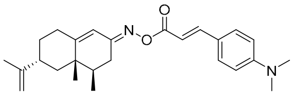	> 20	> 20
N51	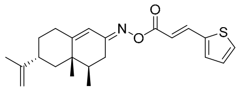	> 20	> 20
N53	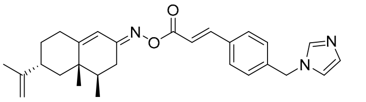	> 20	> 20
Adriamycin	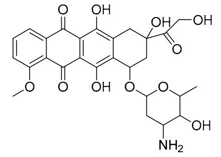	0.30±0.11	0.66±0.11

Note: Adriamycin is a positive drug.
