# Supplementary material for: Nootkatone Derivative Nootkatone-(E)-2-iodobenzoyl hydrazone Promotes Megakaryocytic Differentiation in Erythroleukemia by Targeting JAK2 and Enhancing JAK2/STAT3 and PKCδ/MAPK Crosstalk
Source: Cells. 2024 Dec 26;14(1):10. doi: 10.3390/cells14010010 (PMC11720125; doi:10.3390/cells14010010)
Supplement: Supplementary file 1 [file cells-14-00010-s001.zip › Revised-Table S4.pdf]

Table S4 Results of predictied targets through SwissTargetPrediction.

Target	Common name	Uniprot ID	ChEMBL ID	Target Class	Probability*	Known actives (3D/2D)
Anandamide amidohydrolase	FAAH	O00519	CHEMBL2243	Enzyme	0.109339753	178 / 0
11-beta-hydroxysteroid dehydrogenase 1	HSD11B1	P28845	CHEMBL4235	Enzyme	0.109339753	455 / 0
Cannabinoid receptor 1	CNR1	P21554	CHEMBL218	Family A G protein-coupled receptor	0.109339753	612 / 0
Cannabinoid receptor 2	CNR2	P34972	CHEMBL253	Family A G protein-coupled receptor	0.109339753	569 / 0
Estradiol 17-beta-dehydrogenase 3	HSD17B3	P37058	CHEMBL4234	Enzyme	0.109339753	13 / 0
Cytochrome P450 11B1	CYP11B1	P15538	CHEMBL1908	Cytochrome P450	0.109339753	86 / 0
Tyrosine-protein kinase ITK/TSK	ITK	Q08881	CHEMBL2959	Kinase	0.109339753	37 / 0

Phosphodiesterase 10A	PDE10A	Q9Y233	CHEMBL4409	Phosphodiesterase	0.109339753	373 / 0
Vanilloid receptor	TRPV1	Q8NER1	CHEMBL4794	Voltage-gated ion channel	0.109339753	288 / 0
Monoamine oxidase A	MAOA	P21397	CHEMBL1951	Oxidoreductase	0.109339753	137 / 0
Monoamine oxidase B	MAOB	P27338	CHEMBL2039	Oxidoreductase	0.109339753	282 / 0
Glycine transporter 1	SLC6A9	P48067	CHEMBL2337	Electrochemical transporter	0.109339753	38 / 0
Epoxide hydratase	EPHX2	P34913	CHEMBL2409	Protease	0.109339753	333 / 0
P2X purinoceptor 3	P2RX3	P56373	CHEMBL2998	Ligand-gated ion channel	0.109339753	22 / 0
Vasopressin V2 receptor (by homology)	AVPR2	P30518	CHEMBL1790	Family A G protein-coupled receptor	0.109339753	113 / 0
Vasopressin V1a receptor (by homology)	AVPR1A	P37288	CHEMBL1889	Family A G protein-coupled receptor	0.109339753	136 / 0

Gamma-secretase	PSEN2<br>PSENEN<br>NCSTN<br>APH1A<br>PSEN1<br>APH1B	P49810<br>Q9NZ42<br>Q92542<br>Q96BI3<br>P49768<br>Q8WW43	CHEMBL2094135	Protease	0.109339753	190 / 0
Acetylcholinesterase	ACHE	P22303	CHEMBL220	Hydrolase	0.109339753	95 / 0
Metabotropic glutamate receptor 5	GRM5	P41594	CHEMBL3227	Family C G protein-coupled receptor	0.109339753	385 / 0
Carbonic anhydrase I	CA1	P00915	CHEMBL261	Lyase	0.109339753	170 / 0
Carbonic anhydrase XII	CA12	O43570	CHEMBL3242	Lyase	0.109339753	102 / 0
Carbonic anhydrase IX	CA9	Q16790	CHEMBL3594	Lyase	0.109339753	125 / 0
Epoxide hydrolase 1	EPHX1	P07099	CHEMBL1968	Protease	0.109339753	59 / 0
Cyclooxygenase-2	PTGS2	P35354	CHEMBL230	Oxidoreductase	0.109339753	130 / 0

Orexin receptor 2	HCRTR2	O43614	CHEMBL4792	Family A G protein-coupled receptor	0.109339753	369 / 0
Orexin receptor 1	HCRTR1	O43613	CHEMBL5113	Family A G protein-coupled receptor	0.109339753	325 / 0
Carnitine O-palmitoyltransferase 1, liver isoform	CPT1A	P50416	CHEMBL1293194	Enzyme	0.109339753	33 / 0
Estradiol 17-beta-dehydrogenase 1	HSD17B1	P14061	CHEMBL3181	Enzyme	0.109339753	21 / 0
Proteinase-activated receptor 1	F2R	P25116	CHEMBL3974	Family A G protein-coupled receptor	0.109339753	43 / 0
Cathepsin S	CTSS	P25774	CHEMBL2954	Protease	0.109339753	92 / 0
Cathepsin L	CTSL	P07711	CHEMBL3837	Protease	0.109339753	68 / 0
NAD-dependent deacetylase sirtuin 2	SIRT2	Q8IXJ6	CHEMBL4462	Eraser	0.109339753	64 / 0
Vascular endothelial growth factor receptor 2	KDR	P35968	CHEMBL279	Kinase	0.109339753	256 / 0

Nicotinamide phosphoribosyltransferase	NAMPT	P43490	CHEMBL1744525	Enzyme	0.109339753	39 / 0
6-phosphofructo-2-kinase/fructose-2,6-bisphosphatase 3	PFKFB3	Q16875	CHEMBL2331053	Enzyme	0.109339753	132 / 0
Adenosine A1 receptor (by homology)	ADORA1	P30542	CHEMBL226	Family A G protein-coupled receptor	0.109339753	174 / 0
Adenosine A2a receptor (by homology)	ADORA2A	P29274	CHEMBL251	Family A G protein-coupled receptor	0.109339753	171 / 0
Thioredoxin	TXN	P10599	CHEMBL2010624	Unclassified protein	0.109339753	1 / 0
Dopamine D2 receptor	DRD2	P14416	CHEMBL217	Family A G protein-coupled receptor	0.109339753	194 / 0
Dopamine D4 receptor	DRD4	P21917	CHEMBL219	Family A G protein-coupled receptor	0.109339753	128 / 0
Sphingosine 1-phosphate receptor Edg-1	S1PR1	P21453	CHEMBL4333	Family A G protein-coupled receptor	0.109339753	19 / 0
Polyadenylate-binding protein 1	PABPC1	P11940	CHEMBL1293286	Unclassified protein	0.109339753	5 / 0

Melatonin receptor 1A	MTNR1A	P48039	CHEMBL1945	Family A G protein-coupled receptor	0.109339753	314 / 0
GABA-A receptor; alpha-3/beta-3/gamma-2	GABRB3<br>GABRA3<br>GABRG2	P28472<br>P34903<br>P18507	CHEMBL2094120	Ligand-gated ion channel	0.109339753	49 / 0
GABA-A receptor; alpha-1/beta-3/gamma-2	GABRB3<br>GABRG2<br>GABRA1	P28472<br>P18507<br>P14867	CHEMBL2094121	Ligand-gated ion channel	0.109339753	41 / 0
GABA-A receptor; alpha-2/beta-3/gamma-2	GABRA2<br>GABRB3<br>GABRG2	P47869<br>P28472<br>P18507	CHEMBL2094130	Ligand-gated ion channel	0.109339753	47 / 0
Melatonin receptor 1B	MTNR1B	P49286	CHEMBL1946	Family A G protein-coupled receptor	0.109339753	277 / 0
Protein farnesyltransferase	FNTA<br>FNTB	P49354<br>P49356	CHEMBL2094108	Enzyme	0.109339753	216 / 0
Serine/threonine-protein kinase PIM1	PIM1	P11309	CHEMBL2147	Kinase	0.109339753	65 / 0
5-lipoxygenase activating protein	ALOX5AP	P20292	CHEMBL4550	Other cytosolic protein	0.109339753	366 / 0
Neurokinin 1 receptor	TACR1	P25103	CHEMBL249	Family A G protein-coupled receptor	0.109339753	91 / 0

MAP kinase p38 alpha	MAPK14	Q16539	CHEMBL260	Kinase	0.109339753	332 / 0
Sodium channel protein type IX alpha subunit	SCN9A	Q15858	CHEMBL4296	Voltage-gated ion channel	0.109339753	72 / 0
Prostaglandin E synthase	PTGES	O14684	CHEMBL5658	Enzyme	0.109339753	47 / 0
Elongation of very long chain fatty acids protein 6	ELOVL6	Q9H5J4	CHEMBL5704	Enzyme	0.109339753	25 / 0
Purinergic receptor P2Y12	P2RY12	Q9H244	CHEMBL2001	Family A G protein-coupled receptor	0.109339753	18 / 0
Delta opioid receptor	OPRD1	P41143	CHEMBL236	Family A G protein-coupled receptor	0.109339753	53 / 0
Signal transducer and activator of transcription 3	STAT3	P40763	CHEMBL4026	Transcription factor	0.109339753	24 / 0
CDC7/DBF4 (Cell division cycle 7-related protein kinase/Activator of S phase kinase)	CDC7	O00311	CHEMBL5443	Kinase	0.109339753	62 / 0
Transient receptor potential cation channel subfamily M member 8 (by homology)	TRPM8	Q7Z2W7	CHEMBL1075319	Voltage-gated ion channel	0.109339753	33 / 0

Serotonin 2b (5-HT2b) receptor	HTR2B	P41595	CHEMBL1833	Family A G protein-couple d receptor	0.109339753	13 / 0
Serotonin 2a (5-HT2a) receptor	HTR2A	P28223	CHEMBL224	Family A G protein-couple d receptor	0.109339753	134 / 0
Serotonin 2c (5-HT2c) receptor	HTR2C	P28335	CHEMBL225	Family A G protein-couple d receptor	0.109339753	79 / 0
c-Jun N-terminal kinase 1	MAPK8	P45983	CHEMBL2276	Kinase	0.109339753	95 / 0
Acyl coenzyme A:cholesterol acyltransferase 1	SOAT1	P35610	CHEMBL2782	Enzyme	0.109339753	10 / 0
Serine/threonine-protein kinase WEE1	WEE1	P30291	CHEMBL5491	Kinase	0.109339753	3 / 0
Translocator protein (by homology)	TSPO	P30536	CHEMBL5742	Membrane receptor	0.109339753	157 / 0
Oxytocin receptor	OXTR	P30559	CHEMBL2049	Family A G protein-couple d receptor	0.109339753	73 / 0
Liver glycogen phosphorylase	PYGL	P06737	CHEMBL2568	Enzyme	0.109339753	68 / 0

Voltage-gated calcium channel alpha2/delta subunit 1	CACNA2D1	P54289	CHEMBL1919	Calcium channel auxiliary subunit alpha2delta family	0.109339753	54 / 0
Dopamine D3 receptor	DRD3	P35462	CHEMBL234	Family A G protein-coupled receptor	0.109339753	58 / 0
Neuropeptide Y receptor type 5	NPY5R	Q15761	CHEMBL4561	Family A G protein-coupled receptor	0.109339753	180 / 0
Tyrosine-protein kinase JAK3	JAK3	P52333	CHEMBL2148	Kinase	0.109339753	50 / 0
Tyrosine-protein kinase JAK1	JAK1	P23458	CHEMBL2835	Kinase	0.109339753	50 / 0
Tyrosine-protein kinase JAK2	JAK2	O60674	CHEMBL2971	Kinase	0.109339753	101 / 0
Tyrosine-protein kinase TYK2	TYK2	P29597	CHEMBL3553	Kinase	0.109339753	13 / 0
Butyrylcholinesterase	BCHE	P06276	CHEMBL1914	Hydrolase	0.109339753	30 / 0

Glucocorticoid receptor	NR3C1	P04150	CHEMBL2034	Nuclear receptor	0.109339753	143 / 0
Serotonin 7 (5-HT7) receptor	HTR7	P34969	CHEMBL3155	Family A G protein-coupled receptor	0.109339753	33 / 0
Cytochrome P450 17A1	CYP17A1	P05093	CHEMBL3522	Cytochrome P450	0.109339753	61 / 0
GABA-A receptor; alpha-5/beta-3/gamma-2	GABRB3 GABRG2 GABRA5	P28472 P18507 P31644	CHEMBL2094122	Ligand-gated ion channel	0.109339753	49 / 0
Mu opioid receptor	OPRM1	P35372	CHEMBL233	Family A G protein-coupled receptor	0.109339753	27 / 0
Kappa Opioid receptor	OPRK1	P41145	CHEMBL237	Family A G protein-coupled receptor	0.109339753	47 / 0
Telomerase reverse transcriptase	TERT	O14746	CHEMBL2916	Enzyme	0.109339753	10 / 0
Tankyrase-1	TNKS	O95271	CHEMBL6164	Enzyme	0.109339753	40 / 0
Voltage-gated T-type calcium channel alpha-1H subunit	CACNA1H	O95180	CHEMBL1859	Voltage-gated ion channel	0.109339753	18 / 0

Thromboxane A2 receptor	TBXA2R	P21731	CHEMBL2069	Family A G protein-coupled receptor	0.109339753	15 / 0
Tyrosine-protein kinase LCK	LCK	P06239	CHEMBL258	Kinase	0.109339753	87 / 0
Dual specificity protein phosphatase 3	DUSP3	P51452	CHEMBL2635	Phosphatase	0.109339753	6 / 0
Protein-tyrosine phosphatase 1B	PTPN1	P18031	CHEMBL335	Phosphatase	0.109339753	16 / 0
Dual specificity phosphatase Cdc25A	CDC25A	P30304	CHEMBL3775	Phosphatase	0.109339753	2 / 0
Carbonic anhydrase II	CA2	P00918	CHEMBL205	Lyase	0	211 / 0
Carbonic anhydrase VII	CA7	P43166	CHEMBL2326	Lyase	0	56 / 0
Estrogen receptor beta	ESR2	Q92731	CHEMBL242	Nuclear receptor	0	12 / 0
Beta secretase 2	BACE2	Q9Y5Z0	CHEMBL2525	Protease	0	33 / 0

Adenosine A3 receptor	ADORA3	P0DMS8	CHEMBL256	Family A G protein-coupled receptor	0	48 / 0
Poly [ADP-ribose] polymerase-1	PARP1	P09874	CHEMBL3105	Enzyme	0	152 / 0
Metabotropic glutamate receptor 1	GRM1	Q13255	CHEMBL3772	Family C G protein-coupled receptor	0	60 / 0
P2X purinoceptor 7	P2RX7	Q99572	CHEMBL4805	Ligand-gated ion channel	0	174 / 0
Beta-secretase 1	BACE1	P56817	CHEMBL4822	Protease	0	102 / 0
