# Supplementary material for: Nootkatone Derivative Nootkatone-(E)-2-iodobenzoyl hydrazone Promotes Megakaryocytic Differentiation in Erythroleukemia by Targeting JAK2 and Enhancing JAK2/STAT3 and PKCδ/MAPK Crosstalk
Source: Cells. 2024 Dec 26;14(1):10. doi: 10.3390/cells14010010 (PMC11720125; doi:10.3390/cells14010010)
Supplement: Supplementary file 1 [file cells-14-00010-s001.zip › Revised-Table S5.pdf]

Table S5 The targets of AML from CTD.

Gene Symbol	Gene ID	Disease Name	Disease ID	Direct Evidence	Inference Score
FAS	355	Leukemia, Myeloid, Acute	MESH:D015470	marker/mechanism	76.69
BCL2	596	Leukemia, Myeloid, Acute	MESH:D015470	marker/mechanism	76.06
CASP7	840	Leukemia, Myeloid, Acute	MESH:D015470	marker/mechanism	75.1
KIT	3815	Leukemia, Myeloid, Acute	MESH:D015470	marker/mechanism	71.08
MYC	4609	Leukemia, Myeloid, Acute	MESH:D015470	marker/mechanism	65.01
CCND2	894	Leukemia, Myeloid, Acute	MESH:D015470	marker/mechanism	62.96
STAT3	6774	Leukemia, Myeloid, Acute	MESH:D015470	marker/mechanism	61.22
CDK6	1021	Leukemia, Myeloid, Acute	MESH:D015470	marker/mechanism	56.7

FOXO1	2308	Leukemia, Myeloid, Acute	MESH:D015470	marker/mechanism	56.38
SGK1	6446	Leukemia, Myeloid, Acute	MESH:D015470	marker/mechanism	52.39
TRP53	22059	Leukemia, Myeloid, Acute	MESH:D015470	marker/mechanism	48.72
SPARC	6678	Leukemia, Myeloid, Acute	MESH:D015470	marker/mechanism	47.87
CSF2	1437	Leukemia, Myeloid, Acute	MESH:D015470	marker/mechanism	46.94
AGRN	375790	Leukemia, Myeloid, Acute	MESH:D015470	marker/mechanism	45.87
KMT2A	4297	Leukemia, Myeloid, Acute	MESH:D015470	marker/mechanism	44.37
CSF1R	1436	Leukemia, Myeloid, Acute	MESH:D015470	marker/mechanism	44.12
NPM1	4869	Leukemia, Myeloid, Acute	MESH:D015470	marker/mechanism	43.76

CD44	960	Leukemia, Myeloid, Acute	MESH:D015470	marker/mechanism	43.41
ANXA2	302	Leukemia, Myeloid, Acute	MESH:D015470	marker/mechanism	42.76
CEBPD	1052	Leukemia, Myeloid, Acute	MESH:D015470	marker/mechanism	42.62
DAPK1	1612	Leukemia, Myeloid, Acute	MESH:D015470	marker/mechanism	41.31
HSPB1	3315	Leukemia, Myeloid, Acute	MESH:D015470	marker/mechanism	41.27
CTSH	1512	Leukemia, Myeloid, Acute	MESH:D015470	marker/mechanism	40.9
CEBPA	1050	Leukemia, Myeloid, Acute	MESH:D015470	marker/mechanism	40.23
FLT3	2322	Leukemia, Myeloid, Acute	MESH:D015470	marker/mechanism	40.09
INPP4B	8821	Leukemia, Myeloid, Acute	MESH:D015470	marker/mechanism	39.37

TERT	7015	Leukemia, Myeloid, Acute	MESH:D015470	marker/mechanism	39.36
S100A8	6279	Leukemia, Myeloid, Acute	MESH:D015470	marker/mechanism	39.29
EPHX1	2052	Leukemia, Myeloid, Acute	MESH:D015470	marker/mechanism	39.13
H1-0	3005	Leukemia, Myeloid, Acute	MESH:D015470	marker/mechanism	37.86
MALAT1	378938	Leukemia, Myeloid, Acute	MESH:D015470	marker/mechanism	37.76
RGS2	5997	Leukemia, Myeloid, Acute	MESH:D015470	marker/mechanism	37.62
HGF	3082	Leukemia, Myeloid, Acute	MESH:D015470	marker/mechanism	37.46
ID2	3398	Leukemia, Myeloid, Acute	MESH:D015470	marker/mechanism	37.4
MX1	4599	Leukemia, Myeloid, Acute	MESH:D015470	marker/mechanism	35.48

RUNX1	861	Leukemia, Myeloid, Acute	MESH:D015470	marker/mechanism	35.4
EIF4EBP1	1978	Leukemia, Myeloid, Acute	MESH:D015470	marker/mechanism	35.19
CAPN2	824	Leukemia, Myeloid, Acute	MESH:D015470	marker/mechanism	34.75
KRAS	3845	Leukemia, Myeloid, Acute	MESH:D015470	marker/mechanism	34.66
RUNX3	864	Leukemia, Myeloid, Acute	MESH:D015470	marker/mechanism	34.58
CST3	1471	Leukemia, Myeloid, Acute	MESH:D015470	marker/mechanism	34.51
ANXA4	307	Leukemia, Myeloid, Acute	MESH:D015470	marker/mechanism	34.36
DNMT3A	1788	Leukemia, Myeloid, Acute	MESH:D015470	marker/mechanism	34.26
IL4R	3566	Leukemia, Myeloid, Acute	MESH:D015470	marker/mechanism	34.11

RAC2	5880	Leukemia, Myeloid, Acute	MESH:D015470	marker/mechanism	33.21
MET	4233	Leukemia, Myeloid, Acute	MESH:D015470	marker/mechanism	32.7
ENO2	2026	Leukemia, Myeloid, Acute	MESH:D015470	marker/mechanism	32.11
MN1	4330	Leukemia, Myeloid, Acute	MESH:D015470	marker/mechanism	32.09
S100A10	6281	Leukemia, Myeloid, Acute	MESH:D015470	marker/mechanism	31.92
ANXA6	309	Leukemia, Myeloid, Acute	MESH:D015470	marker/mechanism	31.47
CD9	928	Leukemia, Myeloid, Acute	MESH:D015470	marker/mechanism	31.33
GFI1	2672	Leukemia, Myeloid, Acute	MESH:D015470	marker/mechanism	31.11
ATP1B1	481	Leukemia, Myeloid, Acute	MESH:D015470	marker/mechanism	30.62

DHX15	1665	Leukemia, Myeloid, Acute	MESH:D015470	marker/mechanism	30.53
TCEA2	6919	Leukemia, Myeloid, Acute	MESH:D015470	marker/mechanism	30.26
H1-2	3006	Leukemia, Myeloid, Acute	MESH:D015470	marker/mechanism	30.09
PDE4B	5142	Leukemia, Myeloid, Acute	MESH:D015470	marker/mechanism	30.09
JAK2	3717	Leukemia, Myeloid, Acute	MESH:D015470	marker/mechanism	29.99
PIM2	11040	Leukemia, Myeloid, Acute	MESH:D015470	marker/mechanism	29.61
RASGRP1	10125	Leukemia, Myeloid, Acute	MESH:D015470	marker/mechanism	29.04
MECOM	2122	Leukemia, Myeloid, Acute	MESH:D015470	marker/mechanism	27.78
FHL2	2274	Leukemia, Myeloid, Acute	MESH:D015470	marker/mechanism	27.61

CAPG	822	Leukemia, Myeloid, Acute	MESH:D015470	marker/mechanism	27.21
MLLT10	8028	Leukemia, Myeloid, Acute	MESH:D015470	marker/mechanism	27.01
IDH1	3417	Leukemia, Myeloid, Acute	MESH:D015470	marker/mechanism	26.99
WT1	7490	Leukemia, Myeloid, Acute	MESH:D015470	marker/mechanism	26.4
TRIO	7204	Leukemia, Myeloid, Acute	MESH:D015470	marker/mechanism	26.33
IFI30	10437	Leukemia, Myeloid, Acute	MESH:D015470	marker/mechanism	25.98
LPAR1	1902	Leukemia, Myeloid, Acute	MESH:D015470	marker/mechanism	25.98
LPP	4026	Leukemia, Myeloid, Acute	MESH:D015470	marker/mechanism	25.73
ADCY7	113	Leukemia, Myeloid, Acute	MESH:D015470	marker/mechanism	25.56

ANXA5	308	Leukemia, Myeloid, Acute	MESH:D015470	marker/mechanism	25.22
NTRK3	4916	Leukemia, Myeloid, Acute	MESH:D015470	marker/mechanism	24.75
CTNNA1	1495	Leukemia, Myeloid, Acute	MESH:D015470	marker/mechanism	24.7
ASXL2	55252	Leukemia, Myeloid, Acute	MESH:D015470	marker/mechanism	24.28
CTSZ	1522	Leukemia, Myeloid, Acute	MESH:D015470	marker/mechanism	24.08
IDH2	3418	Leukemia, Myeloid, Acute	MESH:D015470	marker/mechanism	24.05
VOPP1	81552	Leukemia, Myeloid, Acute	MESH:D015470	marker/mechanism	24.02
ZBTB7A	51341	Leukemia, Myeloid, Acute	MESH:D015470	marker/mechanism	23.65
PXDN	7837	Leukemia, Myeloid, Acute	MESH:D015470	marker/mechanism	23.46

RUNX1T1	862	Leukemia, Myeloid, Acute	MESH:D015470	marker/mechanism	23.46
MYH11	4629	Leukemia, Myeloid, Acute	MESH:D015470	marker/mechanism	23.04
GATA2	2624	Leukemia, Myeloid, Acute	MESH:D015470	marker/mechanism	22.94
PSIP1	11168	Leukemia, Myeloid, Acute	MESH:D015470	marker/mechanism	22.27
NRAS	4893	Leukemia, Myeloid, Acute	MESH:D015470	marker/mechanism	22.05
POU4F1	5457	Leukemia, Myeloid, Acute	MESH:D015470	marker/mechanism	21.55
TUBB2A	7280	Leukemia, Myeloid, Acute	MESH:D015470	marker/mechanism	21.46
EHD3	30845	Leukemia, Myeloid, Acute	MESH:D015470	marker/mechanism	21.28
SEPTIN9	10801	Leukemia, Myeloid, Acute	MESH:D015470	marker/mechanism	21.28

SPRY4	81848	Leukemia, Myeloid, Acute	MESH:D015470	marker/mechanism	21.23
TRH	7200	Leukemia, Myeloid, Acute	MESH:D015470	marker/mechanism	21.04
ETV6	2120	Leukemia, Myeloid, Acute	MESH:D015470	marker/mechanism	20.52
EHMT2	10919	Leukemia, Myeloid, Acute	MESH:D015470	marker/mechanism	19.62
SPI1	6688	Leukemia, Myeloid, Acute	MESH:D015470	marker/mechanism	19.29
SVIL	6840	Leukemia, Myeloid, Acute	MESH:D015470	marker/mechanism	19.14
ERG	2078	Leukemia, Myeloid, Acute	MESH:D015470	marker/mechanism	19.11
CBFB	865	Leukemia, Myeloid, Acute	MESH:D015470	marker/mechanism	18.66
BAALC	79870	Leukemia, Myeloid, Acute	MESH:D015470	marker/mechanism	18.28

KMT2C	58508	Leukemia, Myeloid, Acute	MESH:D015470	marker/mechanism	18.24
BACH2	60468	Leukemia, Myeloid, Acute	MESH:D015470	marker/mechanism	18.16
TSC2	7249	Leukemia, Myeloid, Acute	MESH:D015470	marker/mechanism	17.82
SYNGR1	9145	Leukemia, Myeloid, Acute	MESH:D015470	marker/mechanism	17.62
PTPN11	5781	Leukemia, Myeloid, Acute	MESH:D015470	marker/mechanism	17.24
PICALM	8301	Leukemia, Myeloid, Acute	MESH:D015470	marker/mechanism	16.85
GAS2L1	10634	Leukemia, Myeloid, Acute	MESH:D015470	marker/mechanism	16.75
DLEU2	8847	Leukemia, Myeloid, Acute	MESH:D015470	marker/mechanism	16.38
RAC3	5881	Leukemia, Myeloid, Acute	MESH:D015470	marker/mechanism	16.38

NUP98	4928	Leukemia, Myeloid, Acute	MESH:D015470	marker/mechanism	14.62
FXYD6	53826	Leukemia, Myeloid, Acute	MESH:D015470	marker/mechanism	14.56
ENAH	55740	Leukemia, Myeloid, Acute	MESH:D015470	marker/mechanism	14.49
CHMP5	51510	Leukemia, Myeloid, Acute	MESH:D015470	marker/mechanism	14.42
VSIG4	11326	Leukemia, Myeloid, Acute	MESH:D015470	marker/mechanism	14.31
LYL1	4066	Leukemia, Myeloid, Acute	MESH:D015470	marker/mechanism	14.09
ASMTL	8623	Leukemia, Myeloid, Acute	MESH:D015470	marker/mechanism	13.92
HOXA9	3205	Leukemia, Myeloid, Acute	MESH:D015470	marker/mechanism	13.4
CHIC2	26511	Leukemia, Myeloid, Acute	MESH:D015470	marker/mechanism	12.37

NF1	4763	Leukemia, Myeloid, Acute	MESH:D015470	marker/mechanism	11.87
CNR2	1269	Leukemia, Myeloid, Acute	MESH:D015470	marker/mechanism	11.57
GTF2I	2969	Leukemia, Myeloid, Acute	MESH:D015470	marker/mechanism	10.42
SH3GL1	6455	Leukemia, Myeloid, Acute	MESH:D015470	marker/mechanism	10.2
CD33	945	Leukemia, Myeloid, Acute	MESH:D015470	marker/mechanism	7.99
NUP214	8021	Leukemia, Myeloid, Acute	MESH:D015470	marker/mechanism	6.77
TNFSF8	944	Leukemia, Myeloid, Acute	MESH:D015470	marker/mechanism	5.82
TNFSF10	8743	Leukemia, Myeloid, Acute	MESH:D015470	therapeutic	75.22
CSF3	1440	Leukemia, Myeloid, Acute	MESH:D015470	therapeutic	32.85

NECTIN2	5819	Leukemia, Myeloid, Acute	MESH:D015470	therapeutic	19.48
AQP9	366	Leukemia, Myeloid, Acute	MESH:D015470	therapeutic	16.99
PVR	5817	Leukemia, Myeloid, Acute	MESH:D015470	therapeutic	4.9
