# Supplementary material for: Nootkatone Derivative Nootkatone-(E)-2-iodobenzoyl hydrazone Promotes Megakaryocytic Differentiation in Erythroleukemia by Targeting JAK2 and Enhancing JAK2/STAT3 and PKCδ/MAPK Crosstalk
Source: Cells. 2024 Dec 26;14(1):10. doi: 10.3390/cells14010010 (PMC11720125; doi:10.3390/cells14010010)
Supplement: Supplementary file 1 [file cells-14-00010-s001.zip › Revised-Table S6.pdf]

Table S6-1 The targets of AML from DISEASES.

Name	Z-score	Confidence
FLT3	8.1	★★★★☆
RARA	7.7	★★★★☆
RUNX1	7.6	★★★★☆
NPM1	7.5	★★★★☆
CD34	7.3	★★★★☆
CD33	7.2	★★★★☆
TET2	6.8	★★★★☆
DNMT3A	6.8	★★★★☆
CEBPA	6.8	★★★★☆
KIT	6.8	★★★★☆
IDH1	6.7	★★★★☆
ABL1	6.7	★★★★☆
TP53	6.7	★★★★☆
IDH2	6.7	★★★★☆
MLLT3	6.6	★★★★☆
ASXL1	6.6	★★★★☆
RUNX1T1	6.6	★★★★☆
MYC	6.4	★★★★☆
CSF3	6.4	★★★★☆
BCL2	6.4	★★★★☆

CD38	6.4	★★★★☆
ANPEP	6.3	★★★★☆
IL3RA	6.3	★★★★☆
AKT1	6.3	★★★★☆
MYH11	6.2	★★★★☆
WT1	6.2	★★★★☆
KMT2A	6.2	★★★★☆
IL3	6.2	★★★★☆
HOXA9	6.2	★★★★☆
JAK2	6.2	★★★★☆
NUP98	6.1	★★★★☆
CD19	6.1	★★★★☆
PRAM1	6.1	★★★★☆
NRAS	6.1	★★★★☆
SPI1	6.1	★★★★☆
SRSF2	6	★★★★☆
CD7	6	★★★★☆
CBFB	6	★★★★☆
GATA2	6	★★★★☆

Table S6-2 The targets of AML from DISEASES.

Name	Source	Evidence	Confidence

FLT3	MedlinePlus	CURATED	★★★★★
AANAT	MedlinePlus	CURATED	★★★★★
RARA	MedlinePlus	CURATED	★★★★★
KRAS	MedlinePlus	CURATED	★★★★★
AARS1	MedlinePlus	CURATED	★★★★★
PML	MedlinePlus	CURATED	★★★★★
KIT	MedlinePlus	CURATED	★★★★★
STAT5B	MedlinePlus	CURATED	★★★★★
NPM1	MedlinePlus	CURATED	★★★★★
RUNX1	MedlinePlus	CURATED	★★★★★
ABCA3	MedlinePlus	CURATED	★★★★★
ZBTB16	MedlinePlus	CURATED	★★★★★
NRAS	MedlinePlus	CURATED	★★★★★
NUMA1	MedlinePlus	CURATED	★★★★★
MYH11	MedlinePlus	CURATED	★★★★★
RUNX1T1	MedlinePlus	CURATED	★★★★★
CBFB	MedlinePlus	CURATED	★★★★★
