# Supplementary material for: Nootkatone Derivative Nootkatone-(E)-2-iodobenzoyl hydrazone Promotes Megakaryocytic Differentiation in Erythroleukemia by Targeting JAK2 and Enhancing JAK2/STAT3 and PKCδ/MAPK Crosstalk
Source: Cells. 2024 Dec 26;14(1):10. doi: 10.3390/cells14010010 (PMC11720125; doi:10.3390/cells14010010)
Supplement: Supplementary file 1 [file cells-14-00010-s001.zip › Revised-Table S7.pdf]

Table S7 The targets of AML from MalaCards.

Symbol	Description	Category	Score	Evidence	PubMed IDs
CEBPA	CCAAT Enhancer Binding Protein Alpha	Protein Coding	1733.59	Molecular basis known 57	11242107 12661007 12692518 (more)
GATA2	GATA Binding Protein 2	Protein Coding	1432.81	Molecular basis known 57	21892162 25397911 20040766 (more)
DNMT3A	DNA Methyltransferase 3 Alpha	Protein Coding	1333.62	Molecular basis known 57	21067377 21518476 22160010 (more)
JAK2	Janus Kinase 2	Protein Coding	1307.99	Molecular basis known 57	15793561 15858187 16247455 (more)
TERT	Telomerase Reverse Transcriptase	Protein Coding	1081.57	Molecular basis known 57	19147845
FLT3	Fms Related Receptor Tyrosine Kinase 3	Protein Coding	1042.14	Molecular basis known 57	11091200 11290608 11442493 (more)
NPM1	Nucleophosmin 1	Protein Coding	1038.83	Molecular basis known 57	15659725 32581362 19657110
RUNX1	RUNX Family Transcription Factor 1	Protein Coding	1038.48	Molecular basis known 57	20722699 10508512 19357396 (more)

KIT	KIT Proto-Oncogene, Receptor Tyrosine Kinase	Protein Coding	1033.58	Molecular basis known 57	7513208 8589724 9657776 (more)
KRAS	KRAS Proto-Oncogene, GTPase	Protein Coding	1032.17	Molecular basis known 57	2278970 3122217 8955068 (more)
ETV6	ETS Variant Transcription Factor 6	Protein Coding	1013.49	Molecular basis known 57	15806161 12661008
TGM6	Transglutaminase 6	Protein Coding	753.23	Pathogenic 5	24755948
CHIC2	Cysteine Rich Hydrophobic Domain 2	Protein Coding	655.02	Molecular basis known 57	
PICALM	Phosphatidylinositol Binding Clathrin Assembly Protein	Protein Coding	614.43	Molecular basis known 57	
MLLT10	MLLT10 Histone Lysine Methyltransferase DOT1L Cofactor	Protein Coding	607.37	Molecular basis known 57	
NUP214	Nucleoporin 214	Protein Coding	607	Molecular basis known 57	
LPP	LIM Domain Containing Preferred Translocation Partner In Lipoma	Protein Coding	603.5	Molecular basis known 57	

IDH2	Isocitrate Dehydrogenase (NADP(+)) 2	Protein Coding	433.55	Pathogenic 5	20946881 21250968 21596855 (more)
TP53	Tumor Protein P53	Protein Coding	433.54	Pathogenic 5	28492532 20013323 24381225 (more)
NRAS	NRAS Proto-Oncogene, GTPase	Protein Coding	432.8	Pathogenic/Likely pathogenic 5	26619011 2278970 3122217 (more)
NSD1	Nuclear Receptor Binding SET Domain Protein 1	Protein Coding	431.58	Pathogenic 5	15942875
DDX41	DEAD-Box Helicase 41	Protein Coding	431.44	Pathogenic 5	26944477 27133828
SF3B1	Splicing Factor 3b Subunit 1	Protein Coding	430.57	Pathogenic/Likely pathogenic 5	23395771 23634996
IDH1	Isocitrate Dehydrogenase (NADP(+)) 1	Protein Coding	408.51	Pathogenic 5	19657110 20946881 22160010 (more)
SRSF2	Serine And Arginine Rich Splicing Factor 2	Protein Coding	407.83	Pathogenic 5	
FANCD2	FA Complementation Group D2	Protein Coding	406.06	Pathogenic 5	

BCOR	BCL6 Corepressor	Protein Coding	404.76	Pathogenic 5	
DNAJC21	DnaJ Heat Shock Protein Family (Hsp40) Member C21	Protein Coding	404.75	Pathogenic 5	
RTEL1	Regulator Of Telomere Elongation Helicase 1	Protein Coding	404.7	Pathogenic 5	
INSL6	Insulin Like 6	Protein Coding	400	Pathogenic 5	15793561 15858187 16247455 (more)
LOC107303338	3p25 FANCD2 Alu-Mediated Recombination Region	Functional Element	400	Pathogenic 5	
LOC126807619	MED14-Independent Group 3 Enhancer GRCh37_chr5:176696443-176697642	Functional Element	400	Pathogenic 5	
LOC126861451	BRD4-Independent Group 4 Enhancer GRCh37_chr12:11991350-11992549	Functional Element	400	Pathogenic 5	15806161
MFSD11	Major Facilitator Superfamily Domain Containing 11	Protein Coding	400	Pathogenic 5	
RTEL1-TNFRSF6B	RTEL1-TNFRSF6B Readthrough (NMD Candidate)	RNA Gene	400	Pathogenic 5	

KMT2A	Lysine Methyltransferase 2A	Protein Coding	367.09	Gene fusion 58	11579461 8319201 9436917 (more)
MIR126	MicroRNA 126	RNA Gene	360.34	Causal 46	18832181
MIR34B	MicroRNA 34b	RNA Gene	356.21	Causal 46	19258499
MIR204	MicroRNA 204	RNA Gene	355.21	Causal 46	18308931
ERCC6L2	ERCC Excision Repair 6 Like 2	Protein Coding	354.6	Causative germline mutation 58	30936069
MIR320A	MicroRNA 320a	RNA Gene	354.16	Causal 46	19135902
SETBP1	SET Binding Protein 1	Protein Coding	306.84	Causative variation 73	
SH3GL1	SH3 Domain Containing GRB2 Like 1, Endophilin A2	Protein Coding	155.42	Genetic Tests 28	
HOTAIR	HOX Transcript Antisense RNA	RNA Gene	155.77	Experimental evidence: Expression 37	25979172 26261618 26622861 (more)

HOTAIRM1	HOXA Transcript Antisense RNA, Myeloid-Specific 1	RNA Gene	155.71	Experimental evidence: Locus 37	26436590
IRAIN	IGF1R Antisense Imprinted Non-Protein Coding RNA	RNA Gene	155.16	Experimental evidence: Regulation 37	25092925
UCA1	Urothelial Cancer Associated 1	RNA Gene	155.16	Experimental evidence: Regulation 37	26053097
CDKN2B-AS1	CDKN2B Antisense RNA 1	RNA Gene	155.06	Experimental evidence: Regulation 37	18185590 21414664
CCDC26	CCDC26 Long Non-Coding RNA	RNA Gene	155.06	Experimental evidence: Regulation 37	25928165
CCAT1	Colon Cancer Associated Transcript 1	RNA Gene	155.03	Experimental evidence: Regulation 37	26923190
TUG1	Taurine Up-Regulated 1	Protein Coding	154.75	Experimental evidence: Expression 37	29654398
WT1-AS	WT1 Antisense RNA	RNA Gene	154.71	Experimental evidence: Regulation 37	10340388 17940140
MEG3	Maternally Expressed 3	RNA Gene	154.26	Experimental evidence: Regulation 37	19595458

MALAT1	Metastasis Associated Lung Adenocarcinoma Transcript 1	RNA Gene	153.82	Experimental evidence: Expression 37	28713913
TUSC7	Tumor Suppressor Candidate 7	RNA Gene	153.2	Experimental evidence: Expression 37	26345353
MIR223HG	MIR223 Host Gene	RNA Gene	150	Experimental evidence 37	27517498
MIR142	MicroRNA 142	RNA Gene	59.87	Unspecified 46	18478077
MIR155	MicroRNA 155	RNA Gene	59.77	Unspecified 46	18308931
MIR21	MicroRNA 21	RNA Gene	59.75	Unspecified 46	18056805
MIR223	MicroRNA 223	RNA Gene	59.49	Unspecified 46	18056805
MIR23A	MicroRNA 23a	RNA Gene	59.22	Unspecified 46	18056805
MIR125A	MicroRNA 125a	RNA Gene	59.18	Unspecified 46	18056805

MIR27A	MicroRNA 27a	RNA Gene	59.09	Unspecified 46	18056805
MIR22	MicroRNA 22	RNA Gene	59.09	Unspecified 46	18056805
MIR335	MicroRNA 335	RNA Gene	58.8	Unspecified 46	18056805
MIR23B	MicroRNA 23b	RNA Gene	58.77	Unspecified 46	18056805
MIR221	MicroRNA 221	RNA Gene	58.66	Unspecified 46	18056805
MIR30A	MicroRNA 30a	RNA Gene	58.6	Unspecified 46	18478077
MIR15B	MicroRNA 15b	RNA Gene	58.01	Unspecified 46	18478077
MIR151A	MicroRNA 151a	RNA Gene	57.98	Unspecified 46	18478077
MIR34C	MicroRNA 34c	RNA Gene	57.82	Unspecified 46	18478077

MIR130B	MicroRNA 130b	RNA Gene	57.68	Unspecified 46	18056805
MIR331	MicroRNA 331	RNA Gene	57.65	Unspecified 46	18478077
MIR324	MicroRNA 324	RNA Gene	57.22	Unspecified 46	18478077
MIR146A	MicroRNA 146a	RNA Gene	56.04	Unspecified 46	19915715
MIR222	MicroRNA 222	RNA Gene	55.67	Unspecified 46	18056805
MIRLET7B	MicroRNA Let-7b	RNA Gene	55.48	Unspecified 46	18056805
MIR210	MicroRNA 210	RNA Gene	55.4	Unspecified 46	18056805
MIRLET7C	MicroRNA Let-7c	RNA Gene	55.36	Unspecified 46	18056805
MIR182	MicroRNA 182	RNA Gene	55.28	Unspecified 46	18478077

MIR128-1	MicroRNA 128-1	RNA Gene	55.23	Unspecified 46	18056805
MIR424	MicroRNA 424	RNA Gene	55.17	Unspecified 46	18056805
MIR195	MicroRNA 195	RNA Gene	55.06	Unspecified 46	18478077
MIR130A	MicroRNA 130a	RNA Gene	54.91	Unspecified 46	18056805
MIR328	MicroRNA 328	RNA Gene	54.82	Unspecified 46	18478077
MIR199B	MicroRNA 199b	RNA Gene	54.77	Unspecified 46	18056805
MIR181C	MicroRNA 181c	RNA Gene	54.72	Unspecified 46	18478077
MIR326	MicroRNA 326	RNA Gene	54.72	Unspecified 46	18478077
MIRLET7E	MicroRNA Let-7e	RNA Gene	54.7	Unspecified 46	18056805

MIR372	MicroRNA 372	RNA Gene	54.44	Unspecified 46	18478077
MIR30D	MicroRNA 30d	RNA Gene	54.4	Unspecified 46	18478077
MIR27B	MicroRNA 27b	RNA Gene	54.38	Unspecified 46	18056805
MIR451A	MicroRNA 451a	RNA Gene	53.97	Unspecified 46	18056805
MIR184	MicroRNA 184	RNA Gene	53.93	Unspecified 46	18478077
MIR374A	MicroRNA 374a	RNA Gene	53.75	Unspecified 46	18478077
MIR340	MicroRNA 340	RNA Gene	53.66	Unspecified 46	18478077
MIR367	MicroRNA 367	RNA Gene	53.13	Unspecified 46	18478077
	MicroRNA 302d	RNA Gene	52.95	Unspecified 46	18478077

MIR128-2	MicroRNA 128-2	RNA Gene	50	Unspecified 46	18056805
MIR147A	MicroRNA 147a	RNA Gene	50	Unspecified 46	18478077
MIR181A2	MicroRNA 181a-2	RNA Gene	50	Unspecified 46	18478077 17330104
MIR325	MicroRNA 325	RNA Gene	50	Unspecified 46	18478077
