# Supplementary figures and images for: Nootkatone Derivative Nootkatone-(E)-2-iodobenzoyl hydrazone Promotes Megakaryocytic Differentiation in Erythroleukemia by Targeting JAK2 and Enhancing JAK2/STAT3 and PKCδ/MAPK Crosstalk
Source: Cells. 2024 Dec 26;14(1):10. doi: 10.3390/cells14010010 (PMC11720125; doi:10.3390/cells14010010)

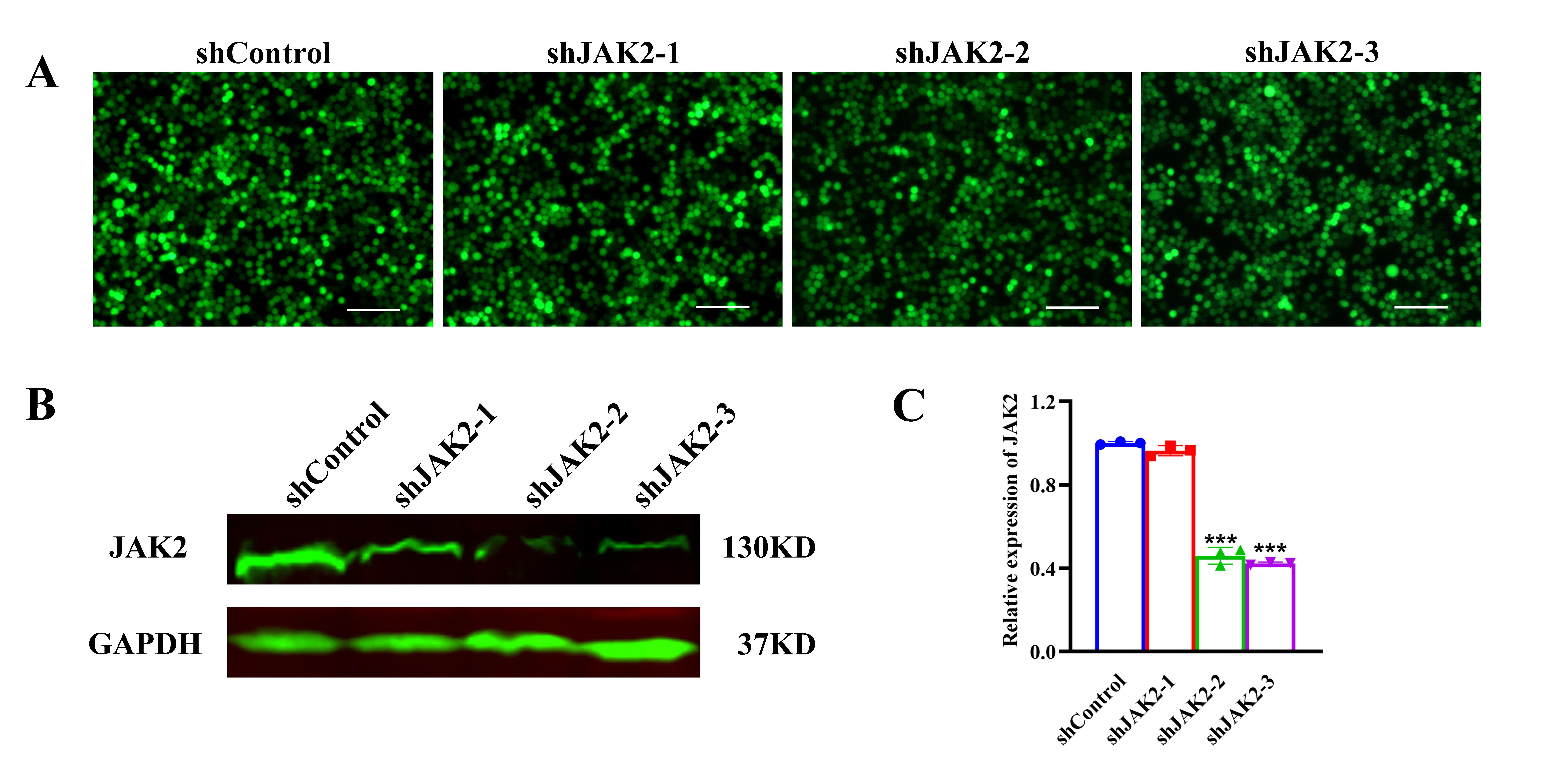

Supplement: Supplementary file 1 [file cells-14-00010-s001.zip › Figure S1.jpg]
