# Supplementary material for: Nootkatone Derivative Nootkatone-(E)-2-iodobenzoyl hydrazone Promotes Megakaryocytic Differentiation in Erythroleukemia by Targeting JAK2 and Enhancing JAK2/STAT3 and PKCδ/MAPK Crosstalk
Source: Cells. 2024 Dec 26;14(1):10. doi: 10.3390/cells14010010 (PMC11720125; doi:10.3390/cells14010010)
Supplement: Supplementary file 1 [file Revised-Table S8.pdf › Revised-Table S8.pdf]

Table S8 The targets of AML from DisGeNET.

Disease	Disease_id	Gene	Gene_id	UniProt	Gene_Full_Name	Protein_Class	N_diseases_g	DSI_g	DPI_g	N_PMIDs	N_SNPs_gda
Childhood Acute Myeloid Leukemia	C0220621	CEBPA	1050	P49715	CCAAT enhancer binding protein alpha	Transcription factor	201	0.496	0.692	4	0
Childhood Acute Myeloid Leukemia	C0220621	FLT3	2322	P36888	fms related receptor tyrosine kinase 3	Kinase	270	0.462	0.654	45	0
Childhood Acute Myeloid Leukemia	C0220621	NPM1	4869	P06748	nucleophosmin 1	Chaperone	283	0.464	0.846	13	0
Childhood Acute Myeloid Leukemia	C0220621	RUNX1	861	Q01196	RUNX family transcription factor 1	Transcription factor	412	0.425	0.808	12	0
Childhood Acute Myeloid Leukemia	C0220621	NUP98	4928	P52948	nucleoporin 98 and 96 precursor	Enzyme	129	0.532	0.577	11	0
Childhood Acute Myeloid Leukemia	C0220621	KMT2A	4297	Q03164	lysine methyltransferase 2A		535	0.41	0.885	21	0
Childhood Acute Myeloid Leukemia	C0220621	KIT	3815	P10721	KIT proto-oncogene, receptor tyrosine kinase	Kinase	715	0.366	0.808	9	0
Childhood Acute Myeloid Leukemia	C0220621	ABCB1	5243	P08183	ATP binding cassette subfamily B member 1	Transporter	933	0.344	0.885	9	0
Childhood Acute Myeloid Leukemia	C0220621	RUNX1T1	862	Q06455	RUNX1 partner transcriptional co-repressor 1	Transcription factor	137	0.53	0.808	9	0
Childhood Acute Myeloid Leukemia	C0220621	WT1	7490	P19544	WT1 transcription factor	Nucleic acid binding	446	0.422	0.808	9	1
Childhood Acute Myeloid Leukemia	C0220621	TBC1D9	23158	Q6ZT07	TBC1 domain family member 9	Enzyme	491	0.399	0.846	7	0
Childhood Acute Myeloid Leukemia	C0220621	NSD1	64324	Q96L73	nuclear receptor binding SET domain protein 1	Epigenetic regulator	247	0.505	0.769	7	0
Childhood Acute Myeloid Leukemia	C0220621	TET2	54790	Q6N021	tet methylcytosine dioxygenase 2		362	0.458	0.808	5	1

Childhood Acute Myeloid Leukemia	C0220621	VEGFA	7422	P15692	vascular endothelial growth factor A	Signaling	1899	0.266	0.923	4	0
Childhood Acute Myeloid Leukemia	C0220621	MIR155	406947		microRNA 155		584	0.384	0.885	4	0
Childhood Acute Myeloid Leukemia	C0220621	BCR	613	P11274	BCR activator of RhoGEF and GTPase	Enzyme modulator	392	0.431	0.885	4	0
Childhood Acute Myeloid Leukemia	C0220621	BAALC	79870	Q8WXS3	BAALC binder of MAP3K1 and KLF4		47	0.631	0.385	3	0
Childhood Acute Myeloid Leukemia	C0220621	TP53	7157	P04637	tumor protein p53	Transcription factor	2494	0.236	0.962	3	0
Childhood Acute Myeloid Leukemia	C0220621	WLS	79971	Q5T9L3	Wnt ligand secretion mediator		166	0.513	0.731	3	0
Childhood Acute Myeloid Leukemia	C0220621	MECOM	2122	Q03112	MDS1 and EVI1 complex locus		191	0.513	0.731	3	0
Childhood Acute Myeloid Leukemia	C0220621	MIR29A	407021		microRNA 29a		278	0.459	0.808	3	0
Childhood Acute Myeloid Leukemia	C0220621	ABCG2	9429	Q9UNQ0	ATP binding cassette subfamily G member 2 (Junior blood group)	Transporter	420	0.419	0.885	3	0
Childhood Acute Myeloid Leukemia	C0220621	IDH2	3418	P48735	isocitrate dehydrogenase (NADP(+)) 2		380	0.434	0.808	3	0
Childhood Acute Myeloid Leukemia	C0220621	MPO	4353	P05164	myeloperoxidase	Enzyme	653	0.377	0.923	3	0
Childhood Acute Myeloid Leukemia	C0220621	ABCC1	4363	P33527	ATP binding cassette subfamily C member 1	Transporter	333	0.445	0.808	3	0
Childhood Acute Myeloid Leukemia	C0220621	CCR7	1236	P32248	C-C motif chemokine receptor 7	G-protein coupled receptor	320	0.443	0.769	3	0
Childhood Acute Myeloid Leukemia	C0220621	PTPN11	5781	Q06124	protein tyrosine phosphatase non-receptor type 11		702	0.385	0.923	3	0
Childhood Acute Myeloid Leukemia	C0220621	CD38	952	P28907	CD38 molecule	Enzyme	473	0.407	0.885	3	0

Childhood Acute Myeloid Leukemia	C0220621	FANCB	2187	Q8NB91	FA complementation group B		278	0.468	0.769	3	0
Childhood Acute Myeloid Leukemia	C0220621	MYH11	4629	P35749	myosin heavy chain 11		161	0.528	0.731	3	0
Childhood Acute Myeloid Leukemia	C0220621	SF3B1	23451	O75533	splicing factor 3b subunit 1	Nucleic acid binding	200	0.511	0.731	2	0
Childhood Acute Myeloid Leukemia	C0220621	IL3	3562	P08700	interleukin 3		211	0.489	0.731	2	0
Childhood Acute Myeloid Leukemia	C0220621	IDH1	3417	O75874	isocitrate dehydrogenase (NADP(+)) 1		399	0.424	0.808	2	0
Childhood Acute Myeloid Leukemia	C0220621	SCT	6343	P09683	secretin		230	0.484	0.846	2	0
Childhood Acute Myeloid Leukemia	C0220621	CXXC5	51523	Q7LFL8	CXXC finger protein 5		35	0.678	0.423	2	0
Childhood Acute Myeloid Leukemia	C0220621	KAT6B	23522	Q8WYB5	lysine acetyltransferase 6B	Enzyme	208	0.525	0.731	2	0
Childhood Acute Myeloid Leukemia	C0220621	PRAME	23532	P78395	preferentially expressed antigen in melanoma		134	0.531	0.577	2	0
Childhood Acute Myeloid Leukemia	C0220621	PRDM16	63976	Q9HAZ2	PR/SET domain 16		200	0.522	0.808	2	0
Childhood Acute Myeloid Leukemia	C0220621	VEGFC	7424	P49767	vascular endothelial growth factor C	Signaling	291	0.459	0.769	2	0
Childhood Acute Myeloid Leukemia	C0220621	MVP	9961	Q14764	major vault protein	Nucleic acid binding	172	0.512	0.769	2	0
Childhood Acute Myeloid Leukemia	C0220621	MARCKSL1	65108	P49006	MARCKS like 1		169	0.508	0.731	2	0
Childhood Acute Myeloid Leukemia	C0220621	CXCR4	7852	P61073	C-X-C motif chemokine receptor 4	G-protein coupled receptor	739	0.362	0.923	2	0
Childhood Acute Myeloid Leukemia	C0220621	ARHGEF1	9138	Q92888	Rho guanine nucleotide exchange factor 1		53	0.623	0.538	2	0

Childhood Acute Myeloid Leukemia	C0220621	ATM	472	Q13315	ATM serine/threonine kinase	Kinase	684	0.374	0.885	2	0
Childhood Acute Myeloid Leukemia	C0220621	GLIS2	84662	Q9BZE0	GLIS family zinc finger 2	Transcription factor	67	0.608	0.462	2	0
Childhood Acute Myeloid Leukemia	C0220621	MIR196B	442920		microRNA 196b		100	0.561	0.731	2	0
Childhood Acute Myeloid Leukemia	C0220621	CSF3	1440	P09919	colony stimulating factor 3		687	0.377	0.885	2	0
Childhood Acute Myeloid Leukemia	C0220621	CBFA2T3	863	O75081	CBFA2/RUNX1 partner transcriptional co-repressor 3	Transcription factor	53	0.638	0.462	2	0
Childhood Acute Myeloid Leukemia	C0220621	CREBBP	1387	Q92793	CREB binding protein		438	0.428	0.808	2	0
Childhood Acute Myeloid Leukemia	C0220621	THPO	7066	P40225	thrombopoietin		185	0.513	0.692	2	0
Childhood Acute Myeloid Leukemia	C0220621	NRAS	4893	P01111	NRAS proto-oncogene, GTPase	Enzyme modulator	611	0.39	0.808	2	0
Childhood Acute Myeloid Leukemia	C0220621	DNMT3A	1788	Q9Y6K1	DNA methyltransferase 3 alpha		350	0.445	0.846	2	0
Childhood Acute Myeloid Leukemia	C0220621	DNMT3B	1789	Q9UBC3	DNA methyltransferase 3 beta		315	0.453	0.846	2	0
Childhood Acute Myeloid Leukemia	C0220621	KAT6A	7994	Q92794	lysine acetyltransferase 6A	Enzyme	129	0.56	0.769	2	0
Childhood Acute Myeloid Leukemia	C0220621	CD34	947	P28906	CD34 molecule		674	0.368	0.808	2	0
Childhood Acute Myeloid Leukemia	C0220621	MDM2	4193	Q00987	MDM2 proto-oncogene	Nucleic acid binding	702	0.362	0.846	2	0
Childhood Acute Myeloid Leukemia	C0220621	CD33	945	P20138	CD33 molecule	Receptor	142	0.524	0.769	2	0
Childhood Acute Myeloid Leukemia	C0220621	IKZF1	10320	Q13422	IKAROS family zinc finger 1	Transcription factor	165	0.533	0.769	2	0

Childhood Acute Myeloid Leukemia	C0220621	NCAM1	4684	P13591	neural cell adhesion molecule 1		445	0.415	0.885	2	0
Childhood Acute Myeloid Leukemia	C0220621	NEO1	4756	Q92859	neogenin 1		60	0.623	0.692	1	0
Childhood Acute Myeloid Leukemia	C0220621	PML	5371	P29590	promyelocytic leukemia		274	0.477	0.846	1	0
Childhood Acute Myeloid Leukemia	C0220621	MTTP	4547	P55157	microsomal triglyceride transfer protein	Transporter	209	0.505	0.846	1	0
Childhood Acute Myeloid Leukemia	C0220621	MIR193B	574455		microRNA 193b		117	0.536	0.808	1	0
Childhood Acute Myeloid Leukemia	C0220621	PTPRA	5786	P18433	protein tyrosine phosphatase receptor type A	Enzyme	91	0.573	0.654	1	0
Childhood Acute Myeloid Leukemia	C0220621	SALL4	57167	Q9UJQ4	spalt like transcription factor 4	Transcription factor	245	0.489	0.769	1	0
Childhood Acute Myeloid Leukemia	C0220621	PCBP4	57060	P57723	poly(rC) binding protein 4	Enzyme	147	0.523	0.808	1	0
Childhood Acute Myeloid Leukemia	C0220621	TRPV2	51393	Q9Y5S1	transient receptor potential cation channel subfamily V member 2	Ion channel	90	0.581	0.615	1	0
Childhood Acute Myeloid Leukemia	C0220621	SBDS	51119	Q9Y3A5	SBDS ribosome maturation factor	Enzyme	105	0.601	0.692	1	0
Childhood Acute Myeloid Leukemia	C0220621	TBX22	50945	Q9Y458	T-box transcription factor 22	Transcription factor	72	0.603	0.577	1	0
Childhood Acute Myeloid Leukemia	C0220621	PAFAH1B1	5048	P43034	platelet activating factor acetylhydrolase 1b regulatory subunit 1		219	0.504	0.769	1	0
Childhood Acute Myeloid Leukemia	C0220621	RTEL1	51750	Q9NZ71	regulator of telomere elongation helicase 1	Enzyme	321	0.459	0.808	1	0
Childhood Acute Myeloid Leukemia	C0220621	MIR382	494331		microRNA 382		71	0.603	0.692	1	0
Childhood Acute Myeloid Leukemia	C0220621	MIR375	494324		microRNA 375		226	0.476	0.769	1	0

Childhood Acute Myeloid Leukemia	C0220621	PLCB4	5332	Q15147	phospholipase C beta 4	Enzyme	89	0.61	0.731	1	0
Childhood Acute Myeloid Leukemia	C0220621	IL17D	53342	Q8TAD2	interleukin 17D		259	0.464	0.808	1	0
Childhood Acute Myeloid Leukemia	C0220621	NTRK1	4914	P04629	neurotrophic receptor tyrosine kinase 1	Kinase	443	0.422	0.808	1	0
Childhood Acute Myeloid Leukemia	C0220621	ASXL2	55252	Q76L83	ASXL transcriptional regulator 2		89	0.617	0.692	1	0
Childhood Acute Myeloid Leukemia	C0220621	PAG1	55824	Q9NWQ8	phosphoprotein membrane anchor with glycosphingolipid microdomains 1		174	0.505	0.808	1	0
Childhood Acute Myeloid Leukemia	C0220621	MAPK3	5595	P27361	mitogen-activated protein kinase 3	Kinase	647	0.379	0.885	1	0
Childhood Acute Myeloid Leukemia	C0220621	RARA	5914	P10276	retinoic acid receptor alpha	Nuclear receptor	274	0.47	0.846	1	0
Childhood Acute Myeloid Leukemia	C0220621	RARRES2	5919	Q99969	retinoic acid receptor responder 2		180	0.519	0.731	1	0
Childhood Acute Myeloid Leukemia	C0220621	OPN1LW	5956	P04000	opsin 1, long wave sensitive	G-protein coupled receptor	197	0.497	0.808	1	0
Childhood Acute Myeloid Leukemia	C0220621	NUP214	8021	P35658	nucleoporin 214	Nucleic acid binding	62	0.638	0.385	1	0
Childhood Acute Myeloid Leukemia	C0220621	MLLT10	8028	P55197	MLLT10 histone lysine methyltransferase DOT1L cofactor	Transcription factor	116	0.551	0.462	1	0
Childhood Acute Myeloid Leukemia	C0220621	CALM2	805	P0DP23;P0DP24;P0DP25	calmodulin 2	Calcium-binding protein	252	0.472	0.808	1	0
Childhood Acute Myeloid Leukemia	C0220621	PTP4A2	8073	Q12974	protein tyrosine phosphatase 4A2		35	0.695	0.423	1	0
Childhood Acute Myeloid Leukemia	C0220621	CALM3	808	P0DP23;P0DP24;P0DP25	calmodulin 3	Calcium-binding protein	215	0.485	0.846	1	0
Childhood Acute Myeloid Leukemia	C0220621	PICALM	8301	Q13492	phosphatidylinositol binding clathrin assembly protein	Transporter	79	0.592	0.538	1	0

Childhood Acute Myeloid Leukemia	C0220621	CASP1	834	P29466	caspase 1	Enzyme	444	0.413	0.885	1	0
Childhood Acute Myeloid Leukemia	C0220621	ZNF382	84911	Q96SR6	zinc finger protein 382	Transcription factor	19	0.76	0.192	1	0
Childhood Acute Myeloid Leukemia	C0220621	RUNX3	864	Q13761	RUNX family transcription factor 3	Transcription factor	281	0.459	0.731	1	0
Childhood Acute Myeloid Leukemia	C0220621	CBFB	865	Q13951	core-binding factor subunit beta		76	0.601	0.538	1	0
Childhood Acute Myeloid Leukemia	C0220621	ABCC3	8714	O15438	ATP binding cassette subfamily C member 3	Transporter	160	0.517	0.769	1	0
Childhood Acute Myeloid Leukemia	C0220621	CES2	8824	O00748	carboxylesterase 2	Enzyme	87	0.584	0.731	1	0
Childhood Acute Myeloid Leukemia	C0220621	SOCS2	8835	O14508	suppressor of cytokine signaling 2	Enzyme modulator	104	0.56	0.731	1	0
Childhood Acute Myeloid Leukemia	C0220621	ABCC10	89845	Q5T3U5	ATP binding cassette subfamily C member 10	Transporter	31	0.682	0.385	1	0
Childhood Acute Myeloid Leukemia	C0220621	USP10	9100	Q14694	ubiquitin specific peptidase 10	Enzyme	40	0.663	0.462	1	0
Childhood Acute Myeloid Leukemia	C0220621	CD6	923	P30203	CD6 molecule	Enzyme	216	0.489	0.808	1	0
Childhood Acute Myeloid Leukemia	C0220621	CD19	930	P15391	CD19 molecule		365	0.43	0.885	1	0
Childhood Acute Myeloid Leukemia	C0220621	ASRGL1	80150	Q7L266	asparaginase and isoaspartyl peptidase 1	Enzyme	205	0.494	0.808	1	0
Childhood Acute Myeloid Leukemia	C0220621	CALM1	801	P0DP23;P0DP24;P0DP25	calmodulin 1	Calcium-binding protein	253	0.472	0.808	1	0
Childhood Acute Myeloid Leukemia	C0220621	TFPI2	7980	P48307	tissue factor pathway inhibitor 2	Enzyme modulator	161	0.521	0.769	1	0
Childhood Acute Myeloid Leukemia	C0220621	BCL2	596	P10415	BCL2 apoptosis regulator	Signaling	1456	0.291	0.885	1	0

Childhood Acute Myeloid Leukemia	C0220621	BCL3	602	P20749	BCL3 transcription coactivator		136	0.541	0.731	1	0
Childhood Acute Myeloid Leukemia	C0220621	SRSF2	6427	Q01130	serine and arginine rich splicing factor 2	Nucleic acid binding	102	0.561	0.731	1	0
Childhood Acute Myeloid Leukemia	C0220621	NOM1	64434	Q5C9Z4	nucleolar protein with MIF4G domain 1	Enzyme	3	0.89	0.192	1	0
Childhood Acute Myeloid Leukemia	C0220621	UCA1	652995		urothelial cancer associated 1		152	0.512	0.808	1	0
Childhood Acute Myeloid Leukemia	C0220621	SLC25A1	6576	P53007	solute carrier family 25 member 1	Transporter	168	0.553	0.731	1	0
Childhood Acute Myeloid Leukemia	C0220621	STAT3	6774	P40763	signal transducer and activator of transcription 3	Nucleic acid binding	1193	0.32	0.923	1	0
Childhood Acute Myeloid Leukemia	C0220621	TERF1	7013	P54274	telomeric repeat binding factor 1		102	0.563	0.692	1	0
Childhood Acute Myeloid Leukemia	C0220621	TGFB1	7040	P01137	transforming growth factor beta 1	Signaling	1558	0.287	0.962	1	0
Childhood Acute Myeloid Leukemia	C0220621	FRTS1	65211		Fanconi renotubular syndrome		69	0.599	0.654	1	0
Childhood Acute Myeloid Leukemia	C0220621	TLR2	7097	O60603	toll like receptor 2		749	0.361	0.923	1	0
Childhood Acute Myeloid Leukemia	C0220621	TLR4	7099	O00206	toll like receptor 4		1174	0.321	0.962	1	0
Childhood Acute Myeloid Leukemia	C0220621	MIR663A	724033		microRNA 663a		81	0.576	0.769	1	0
Childhood Acute Myeloid Leukemia	C0220621	U2AF1	7307	P0DN76;Q01081	U2 small nuclear RNA auxiliary factor 1	Nucleic acid binding	67	0.604	0.577	1	0
Childhood Acute Myeloid Leukemia	C0220621	YWHAE	7531	P62258	tyrosine 3-monooxygenase/tryptophan 5-monooxygenase activation protein epsilon	Chaperone	176	0.53	0.692	1	0
Childhood Acute Myeloid Leukemia	C0220621	DEK	7913	P35659	DEK proto-oncogene		122	0.542	0.769	1	0

Childhood Acute Myeloid Leukemia	C0220621	DERL1	79139	Q9BUN8	derlin 1	Receptor	85	0.576	0.654	1	0
Childhood Acute Myeloid Leukemia	C0220621	SNAP91	9892	O60641	synaptosome associated protein 91	Transporter	53	0.638	0.538	1	0
Childhood Acute Myeloid Leukemia	C0220621	GSN	2934	P06396	gelsolin	Cellular structure	262	0.475	0.885	1	0
Childhood Acute Myeloid Leukemia	C0220621	SEPTIN6	23157	Q14141	septin 6	Enzyme modulator	41	0.644	0.385	1	0
Childhood Acute Myeloid Leukemia	C0220621	KLRK1	22914	P26718	killer cell lectin like receptor K1		255	0.467	0.769	1	0
Childhood Acute Myeloid Leukemia	C0220621	ETV6	2120	P41212	ETS variant transcription factor 6	Transcription factor	241	0.477	0.731	1	0
Childhood Acute Myeloid Leukemia	C0220621	ABCA3	21	Q99758	ATP binding cassette subfamily A member 3	Transporter	84	0.592	0.615	1	0
Childhood Acute Myeloid Leukemia	C0220621	ERG	2078	P11308	ETS transcription factor ERG	Transcription factor	298	0.453	0.808	1	0
Childhood Acute Myeloid Leukemia	C0220621	ERCC2	2068	P18074	ERCC excision repair 2, TFIIH core complex helicase subunit	Enzyme	499	0.42	0.846	1	1
Childhood Acute Myeloid Leukemia	C0220621	ELANE	1991	P08246	elastase, neutrophil expressed	Enzyme	346	0.447	0.846	1	0
Childhood Acute Myeloid Leukemia	C0220621	EIF4E	1977	P06730	eukaryotic translation initiation factor 4E	Nucleic acid binding	317	0.448	0.846	1	0
Childhood Acute Myeloid Leukemia	C0220621	EIF4A2	1974	Q14240	eukaryotic translation initiation factor 4A2		74	0.604	0.731	1	0
Childhood Acute Myeloid Leukemia	C0220621	BRD4	23476	O60885	bromodomain containing 4	Epigenetic regulator	335	0.446	0.885	1	0
Childhood Acute Myeloid Leukemia	C0220621	IL27	246778	Q8NEV9	interleukin 27		286	0.457	0.808	1	0
Childhood Acute Myeloid Leukemia	C0220621	ABL1	25	P00519	ABL proto-oncogene 1, non-receptor tyrosine kinase	Kinase	395	0.432	0.923	1	0

Childhood Acute Myeloid Leukemia	C0220621	GLI1	2735	P08151	GLI family zinc finger 1		369	0.431	0.846	1	0
Childhood Acute Myeloid Leukemia	C0220621	HPGDS	27306	O60760	hematopoietic prostaglandin D synthase		570	0.388	0.923	1	0
Childhood Acute Myeloid Leukemia	C0220621	GATA4	2626	P43694	GATA binding protein 4		336	0.458	0.846	1	0
Childhood Acute Myeloid Leukemia	C0220621	GATA2	2624	P23769	GATA binding protein 2		229	0.496	0.654	1	0
Childhood Acute Myeloid Leukemia	C0220621	GATA1	2623	P15976	GATA binding protein 1		264	0.483	0.808	1	0
Childhood Acute Myeloid Leukemia	C0220621	SETBP1	26040	Q9Y6X0	SET binding protein 1		257	0.503	0.808	1	0
Childhood Acute Myeloid Leukemia	C0220621	CCDC28A	25901	Q8IWP9	coiled-coil domain containing 28A	Receptor	10	0.78	0.154	1	0
Childhood Acute Myeloid Leukemia	C0220621	EBF3	253738	Q9H4W6	EBF transcription factor 3		124	0.595	0.731	1	0
Childhood Acute Myeloid Leukemia	C0220621	FUS	2521	P35637	FUS RNA binding protein	Nucleic acid binding	301	0.47	0.846	1	0
Childhood Acute Myeloid Leukemia	C0220621	EIF4A1	1973	P60842	eukaryotic translation initiation factor 4A1		57	0.631	0.654	1	0
Childhood Acute Myeloid Leukemia	C0220621	ATN1	1822	P54259	atrophin 1		499	0.409	0.885	1	0
Childhood Acute Myeloid Leukemia	C0220621	ASXL1	171023	Q8IXJ9	ASXL transcriptional regulator 1		296	0.482	0.808	1	0
Childhood Acute Myeloid Leukemia	C0220621	MLLT11	10962	Q13015	MLLT11 transcription factor 7 cofactor		58	0.633	0.5	1	0
Childhood Acute Myeloid Leukemia	C0220621	EBP	10682	Q15125	EBP cholestenol delta-isomerase	Enzyme	243	0.494	0.846	1	0
Childhood Acute Myeloid Leukemia	C0220621	LINC02682	105376570		long intergenic non-protein coding RNA 2682		3	1	0.038	1	0

Childhood Acute Myeloid Leukemia	C0220621	NCOA2	10499	Q15596	nuclear receptor coactivator 2	Enzyme	95	0.575	0.615	1	0
Childhood Acute Myeloid Leukemia	C0220621	EFS	10278	O43281	embryonal Fyn-associated substrate		75	0.597	0.615	1	0
Childhood Acute Myeloid Leukemia	C0220621	U2AF1L5	102724594	P0DN76;Q01081	U2 small nuclear RNA auxiliary factor 1 like 5		14	0.769	0.308	1	0
Childhood Acute Myeloid Leukemia	C0220621	CEBPZ	10153	Q03701	CCAAT enhancer binding protein zeta	Transcription factor	76	0.595	0.692	1	0
Childhood Acute Myeloid Leukemia	C0220621	ABCB6	10058	Q9NP58	ATP binding cassette subfamily B member 6 (Langereis blood group)	Transporter	400	0.431	0.885	1	0
Childhood Acute Myeloid Leukemia	C0220621	KLRC4-KLRK1	100528032	P26718	KLRC4-KLRK1 readthrough		246	0.469	0.769	1	0
Childhood Acute Myeloid Leukemia	C0220621	PIM2	11040	Q9P1W9	Pim-2 proto-oncogene, serine/threonine kinase	Kinase	68	0.606	0.538	1	0
Childhood Acute Myeloid Leukemia	C0220621	PSIP1	11168	O75475	PC4 and SFRS1 interacting protein 1	Signaling	210	0.49	0.846	1	0
Childhood Acute Myeloid Leukemia	C0220621	RHEBL1	121268	Q8TAI7	RHEB like 1	Enzyme modulator	5	0.89	0.115	1	0
Childhood Acute Myeloid Leukemia	C0220621	DDX10	1662	Q13206	DEAD-box helicase 10		22	0.722	0.269	1	0
Childhood Acute Myeloid Leukemia	C0220621	ZNF296	162979	Q8WUU4	zinc finger protein 296	Transcription factor	11	0.769	0.115	1	0
Childhood Acute Myeloid Leukemia	C0220621	CTSL	1514	P07711	cathepsin L	Enzyme	225	0.488	0.846	1	0
Childhood Acute Myeloid Leukemia	C0220621	CSF3R	1441	Q99062	colony stimulating factor 3 receptor	Signaling	120	0.55	0.692	1	0
Childhood Acute Myeloid Leukemia	C0220621	CSF2	1437	P04141	colony stimulating factor 2	Signaling	1028	0.33	0.962	1	0
Childhood Acute Myeloid Leukemia	C0220621	CSF1R	1436	P07333	colony stimulating factor 1 receptor	Kinase	356	0.448	0.808	1	0

Childhood Acute Myeloid Leukemia	C0220621	CCDC26	137196	Q8TAB7	CCDC26 long non-coding RNA		60	0.647	0.615	1	0
Childhood Acute Myeloid Leukemia	C0220621	CPOX	1371	P36551	coproporphyrinogen oxidase	Enzyme	246	0.488	0.846	1	0
Childhood Acute Myeloid Leukemia	C0220621	CNTFR	1271	P26992	ciliary neurotrophic factor receptor	Signaling	19	0.722	0.385	1	0
Childhood Acute Myeloid Leukemia	C0220621	PDCD6	10016	O75340	programmed cell death 6		45	0.653	0.5	1	0
Childhood Acute Myeloid Leukemia	C0220621	GSTM1	2944	P09488	glutathione S-transferase mu 1		627	0.38	0.923	1	0
Childhood Acute Myeloid Leukemia	C0220621	ND3	4537	P03897	NADH dehydrogenase, subunit 3 (complex I)		113	0.604	0.654	1	0
Childhood Acute Myeloid Leukemia	C0220621	MIR34A	407040		microRNA 34a		428	0.421	0.846	1	0
Childhood Acute Myeloid Leukemia	C0220621	MIR29C	407026		microRNA 29c		183	0.496	0.808	1	0
Childhood Acute Myeloid Leukemia	C0220621	MIR25	407014		microRNA 25		148	0.517	0.769	1	0
Childhood Acute Myeloid Leukemia	C0220621	MIR206	406989		microRNA 206		226	0.477	0.846	1	0
Childhood Acute Myeloid Leukemia	C0220621	MIR195	406971		microRNA 195		180	0.496	0.769	1	0
Childhood Acute Myeloid Leukemia	C0220621	MIR192	406967		microRNA 192		173	0.503	0.846	1	0
Childhood Acute Myeloid Leukemia	C0220621	MIR183	406959		microRNA 183		190	0.494	0.885	1	0
Childhood Acute Myeloid Leukemia	C0220621	MIR146A	406938		microRNA 146a		505	0.398	0.885	1	0
Childhood Acute Myeloid Leukemia	C0220621	MIR122	406906		microRNA 122		250	0.468	0.808	1	0

Childhood Acute Myeloid Leukemia	C0220621	MIR34B	407041		microRNA 34b		165	0.509	0.808	1	0
Childhood Acute Myeloid Leukemia	C0220621	MDM4	4194	O15151	MDM4 regulator of p53	Nucleic acid binding	265	0.461	0.808	1	0
Childhood Acute Myeloid Leukemia	C0220621	MEF2C	4208	Q06413	myocyte enhancer factor 2C	Transcription factor	206	0.517	0.769	1	0
Childhood Acute Myeloid Leukemia	C0220621	ATP6	4508	P00846	ATP synthase F0 subunit 6	Transporter	226	0.541	0.731	1	0
Childhood Acute Myeloid Leukemia	C0220621	MT3	4504	P25713	metallothionein 3		108	0.582	0.615	1	0
Childhood Acute Myeloid Leukemia	C0220621	MSH3	4437	P20585	mutS homolog 3	Nucleic acid binding	245	0.474	0.769	1	0
Childhood Acute Myeloid Leukemia	C0220621	MIR370	442915		microRNA 370		112	0.546	0.808	1	0
Childhood Acute Myeloid Leukemia	C0220621	MIR342	442909		microRNA 342		111	0.547	0.769	1	0
Childhood Acute Myeloid Leukemia	C0220621	MIR335	442904		microRNA 335		141	0.521	0.808	1	0
Childhood Acute Myeloid Leukemia	C0220621	MN1	4330	Q10571	MN1 proto-oncogene, transcriptional regulator		47	0.682	0.385	1	0
Childhood Acute Myeloid Leukemia	C0220621	MME	4311	P08473	membrane metalloendopeptidase	Enzyme	470	0.407	0.808	1	0
Childhood Acute Myeloid Leukemia	C0220621	AFDN	4301	P55196	afadin, adherens junction formation factor	Cellular structure	46	0.65	0.462	1	0
Childhood Acute Myeloid Leukemia	C0220621	MIR10B	406903		microRNA 10b		167	0.509	0.808	1	0
Childhood Acute Myeloid Leukemia	C0220621	MIR100	406892		microRNA 100		146	0.521	0.846	1	0
Childhood Acute Myeloid Leukemia	C0220621	LRP1	4035	Q07954	LDL receptor related protein 1		252	0.482	0.769	1	0

Childhood Acute Myeloid Leukemia	C0220621	FAS	355	P25445	Fas cell surface death receptor		754	0.372	0.923	1	0
Childhood Acute Myeloid Leukemia	C0220621	IGFBP7	3490	Q16270	insulin like growth factor binding protein 7		241	0.476	0.769	1	0
Childhood Acute Myeloid Leukemia	C0220621	IFNG	3458	P01579	interferon gamma		1519	0.288	0.962	1	0
Childhood Acute Myeloid Leukemia	C0220621	HTC2	3342		hypertrichosis 2 (generalized, congenital)		511	0.392	0.808	1	0
Childhood Acute Myeloid Leukemia	C0220621	BIRC5	332	O15392	baculoviral IAP repeat containing 5	Enzyme modulator	199	0.495	0.731	1	0
Childhood Acute Myeloid Leukemia	C0220621	XIAP	331	P98170	X-linked inhibitor of apoptosis	Enzyme modulator	321	0.447	0.808	1	0
Childhood Acute Myeloid Leukemia	C0220621	HOXD10	3236	P28358	homeobox D10		83	0.59	0.538	1	0
Childhood Acute Myeloid Leukemia	C0220621	MNX1	3110	P50219	motor neuron and pancreas homeobox 1	Transcription factor	113	0.555	0.615	1	0
Childhood Acute Myeloid Leukemia	C0220621	HDAC1	3065	Q13547	histone deacetylase 1	Epigenetic regulator	277	0.46	0.846	1	0
Childhood Acute Myeloid Leukemia	C0220621	IL2	3558	P60568	interleukin 2		950	0.336	0.885	1	0
Childhood Acute Myeloid Leukemia	C0220621	IL2RG	3561	P31785	interleukin 2 receptor subunit gamma	Signaling	164	0.535	0.769	1	0
Childhood Acute Myeloid Leukemia	C0220621	IL3RA	3563	P26951	interleukin 3 receptor subunit alpha	Signaling	95	0.56	0.769	1	0
Childhood Acute Myeloid Leukemia	C0220621	CASC15	401237		cancer susceptibility 15		90	0.603	0.577	1	0
Childhood Acute Myeloid Leukemia	C0220621	RPSA	3921	P08865	ribosomal protein SA	Nucleic acid binding	221	0.485	0.769	1	0
Childhood Acute Myeloid Leukemia	C0220621	KRAS	3845	P01116	KRAS proto-oncogene, GTPase	Enzyme modulator	1213	0.32	0.923	1	0

Childhood Acute Myeloid Leukemia	C0220621	KCNH2	3757	Q12809	potassium voltage-gated channel subfamily H member 2	Ion channel	189	0.522	0.731	1	3
Childhood Acute Myeloid Leukemia	C0220621	CD82	3732	P27701	CD82 molecule		172	0.507	0.731	1	0
Childhood Acute Myeloid Leukemia	C0220621	JAK2	3717	O60674	Janus kinase 2	Kinase	644	0.385	0.885	1	0
Childhood Acute Myeloid Leukemia	C0220621	ITGAL	3683	P20701	integrin subunit alpha L		169	0.511	0.885	1	0
Childhood Acute Myeloid Leukemia	C0220621	IDO1	3620	P14902	indoleamine 2,3-dioxygenase 1		295	0.454	0.846	1	0
Childhood Acute Myeloid Leukemia	C0220621	IL6	3569	P05231	interleukin 6		2367	0.248	0.962	1	0
Childhood Acute Myeloid Leukemia	C0220621	ANXA2	302	P07355	annexin A2		294	0.453	0.808	1	0
